# Long-Term Effects of Stress During Adolescence on the Sex-Dependent Responses of Thyroid Axis and Target Tissues to Exercise in Male and Female Wistar Rats

**DOI:** 10.3390/ijms26199425

**Published:** 2025-09-26

**Authors:** Marco Parra-Montes de Oca, Lorraine Jaimes-Hoy, Karen Garduño, Rodrigo García-Herrera, Jean-Louis Charli, Patricia Joseph-Bravo

**Affiliations:** 1 Departamento de Genética del Desarrollo y Fisiología Molecular, Instituto de Biotecnología, Universidad Nacional Autónoma de México (UNAM), Avenida Universidad 2001, Colonia Chamilpa, Cuernavaca 62210, Morelos, Mexico; marcopm@live.com.mx (M.P.-M.d.O.); lorraine.jaimes@ibt.unam.mx (L.J.-H.); karengm@ciencias.unam.mx (K.G.); charli.casalonga@ibt.unam.mx (J.-L.C.); 2 Redes por la Diversidad, Equidad y Sustentabilidad, A.C., Cuetzalan 73560, Puebla, Mexico; rgarcia@riseup.net

**Keywords:** HPT axis, HPA axis, chronic variable stress, exercise, calorie restriction, adolescence, sex differences, skeletal muscle, white adipose tissue, brown adipose tissue

## Abstract

The response of the hypothalamic–pituitary–thyroid (HPT) axis to energy demands is perturbed by previous chronic stress perceived during the neonatal or adult periods. We examined the effects of chronic variable stress (CVS) during adolescence on the responses of the HPT axis and target tissues of adult rats to 14 days of voluntary wheel running (Ex) or pair-feeding (PF) to match the reduced food intake of exercised rats. CVS increased the expression of *Gr* in the paraventricular nucleus (PVN) and of *Npy* in the mediobasal hypothalamus (MBH) in males; serum corticosterone concentration increased (1.5×), MBH *Dio2* and PVN *Trh* decreased (40%) in both sexes, serum fT4 increased only in males, while T3 and fT3 increased (2×) in females. Exercise decreased Cort and increased PVN *Trh* expression only in males. In both sexes, it increased MBH *Pomc* and *Dio2* (2×), skeletal muscle *Dio2* and *Pgc1a* (2×), inguinal and perigonadal white adipose tissue (WAT) *Adrb3*, *Dio2*, *Pparg*, *Hsl* (1.5×), and brown adipose tissue *Adrb3*, *Dio2*, and *Ucp1*. All exercise-induced changes were repressed in CVS-Ex, except *Hsl* in inguinal WAT of both sexes, or BAT *Dio2* in females, which, in contrast, was stimulated (1.5×). PF had lower values than sedentary in most parameters. These results support the idea that adolescent stress affects adult metabolic and neuroendocrine responses to exercise in a sex-specific manner.

## 1. Introduction

Modern societies bear the burden of a wide range of metabolic, cardiovascular, and psychological pathologies, many of which stem from a sedentary lifestyle and/or chronic stress, even in the early stages of development [1,2,3]. Stress, defined as the reaction to “challenges, real or implied, to the homeostatic regulatory processes of the organism”, immediately stimulates the autonomic nervous system and, within minutes, activates the HPA axis [2]. Whether stressors are physical or psychological, the physiological response depends largely on their controllability and, in the case of chronic exposure, on the individual’s ability to adapt [3]. The type, intensity and timing of stressor exposure critically shape the outcome: chronic stress can elicit hyper- or hypo-activation of the hypothalamic–pituitary–adrenal (HPA) axis. Habituation usually occurs with repeated exposure to homotypic stressors, whereas heterotypic stressors tend to provoke sustained hyperactivation of HPA. Failure to cope with chronic stress and adapt adequately leads to dysregulation and pathologies [2,3,4]. Chronic stress has been implicated in various metabolic and psychiatric disorders, especially when experienced during critical developmental periods, such as the perinatal stage or adolescence, leading to long-lasting effects [4,5,6]. Regular physical exercise improves metabolism and alleviates stress-related pathologies, such as depression, contributing to disease prevention or mitigation [7,8]. Exercise performance requires an immediate and adequate supply of energy, which depends on concerted and efficient responses of the somatic, sympathetic, and neuroendocrine systems, which communicate with the participating organs through neurotransmitters, hormones, and exerkines [7,8,9].

The hypothalamus serves as the central integrator of energy homeostasis, decoding nutritional and psychological signals and generating behavioral, sympathetic, and endocrine responses [10]. The neurons in the arcuate nucleus (Arc) expressing pro-opiomelanocortin (POMC) or Agouti-related peptide/neuropeptide Y (AgRP/NPY) sense the energetic state and project to the paraventricular nucleus (PVN), which relays signals through multisynaptic connections to the brain stem and sympathetic neurons targeting peripheral organs [11,12,13]. At the PVN level, hypophysiotropic neurons synthesize thyrotropin-releasing hormone (TRH) or corticotropin-releasing hormone (CRH), controlling hypothalamus–pituitary–thyroid (HPT: TRH: thyrotropin (TSH): T4, T3) [14,15] or hypothalamus–pituitary–adrenal (HPA: corticotrophin-releasing hormone (CRH): corticotrophin (ACTH): cortisol/corticosterone (Cort)) axes [16] (Supplementary Figure S1). T3 and glucocorticoids exert feedback control at the hypothalamic and pituitary levels [14,15,16,17], are modulated by circadian cycles, regulate carbohydrate and lipid metabolism, and mobilize substrates to oxidizing tissues [17,18]. Most of T3, the transcriptionally active hormone, is formed by deiodinases that act on T4 released from the thyroid; DIO2 is exquisitely regulated in a tissue-specific manner, setting the concentration of active T3 in situ [19,20]. Thyroid hormones (TH) interact with adrenergic signaling in a coordinated or synergistic fashion in white and brown adipose tissues (WAT, BAT), where they activate lipolysis, de novo lipogenesis, or thermogenesis [21,22,23] and are essential in mitochondrial biogenesis by activating peroxisome proliferator-activated receptor γ coactivator 1α (PGC1-α) [24,25]. TH participates in the adequate functioning of the nervous and cardiorespiratory systems [22,26].

The activity of the HPT axis is inhibited in conditions of energy deficit and by several types of acute or chronic stress (reviewed in [27,28]), except by physical stressors that demand energy, such as cold exposure or exercise [29,30,31]. Rapid and transient increases in PVN TRH expression and serum thyroid-stimulating hormone (TSH) concentration during the first hour of cold exposure or increased physical activity are followed by increased T4 or T3 concentration in the circulation [30,31]; responses to intense exercise or longer exposure times depend on the energy reserves of the individual [32]. The response of the HPA or HPT axes to a new challenge may be altered by previous stress exposures, even if some of them date from critical periods of development [33,34,35]. In the case of previous acute stress, some effects can be attributed to the dynamics of corticosterone-induced activation of the glucocorticoid receptor (GR) that blunts a subsequent response of the HPA axis if, for example, corticosterone is administered to male rats 3 h before restraint [36], or before cold exposure that blunts the response of the HPT axis [37,38]. We showed that after two weeks of exposure to homotypic stress (restraint) or heterotypic stress (chronic variable stress [CVS]), rats do not show increases in PVN *Trh* expression and serum TSH concentration induced by cold, their body temperature decreases, and cold-induced BAT thermogenesis is absent, while in the same animals, the HPA is stimulated only in rats previously submitted to CVS [39]. These cold responses are also disrupted in male adult rats previously stressed during critical developmental periods, such as those imposed by maternal separation, which causes altered HPA axis reactivity in adults [35].

The ameliorative effects of exercise on stress-induced disturbances are actively investigated, with most research focusing on behavior and the effects of exercise performed before or concurrently with stress exposure [40]; fewer studies address the immediate or long-term effects of chronic stress prior to exercise on performance and plasticity [41]. We previously reported that rats exposed to stress for two weeks prior to being exposed to voluntary wheel running (WR) reduce the total distance traveled, the amount of fat loss, and inhibit some HPT axis responses induced in control adult rats, which is different in males and females [42]. The long-term effects of maternal separation also interfere with the response of the HPT axis to WR as adults, more in females than males [35].

Because the effects produced in the elements of the HPT axis are small, we hypothesized that the changes would be more easily detected by measuring the expression of molecules relevant to T3 action in target organs such as skeletal muscle (SKM), perigonadal WAT (pgWAT), inguinal WAT (iWAT), and brown adipose tissue (BAT). Voluntary wheel running was chosen because it is a less stressful paradigm compared to other types of aerobic exercise, such as treadmill or swimming; it has the motivation component that can be affected by stress or metabolic differences [43,44] and has been shown to reduce depression in several behavioral animal models [45]. The disadvantage of voluntary running is that proper kinetic measurements cannot be performed since rats do not run continuously but in bouts, and more intensely during the first hours of the dark period [46,47]; however, since we have shown that the response of the HPT axis is fast and transient, we prefer to evaluate the animals in their post-exercise adaptation period [48] (3 h after light change) to be able to detect changes due to the cumulative periods of intermittent running during the active time of 14 days [49].

In this work, we examine how chronic stress during adolescence affects neuroendocrine and metabolic responses to exercise in male and female adult rats. Adolescence was chosen since this is when the HPA matures [50,51] and when long-term effects are reported in females, who are more resistant to stress at other stages of life [52,53,54]. We chose the CVS paradigm to avoid habituation, a paradigm that has previously been shown to alter basal and cold-induced activation of the HPT axis [39,55].

## 2. Results

### 2.1. Behavioral and Ponderal Variables During Adolescence

**Figure 1:**
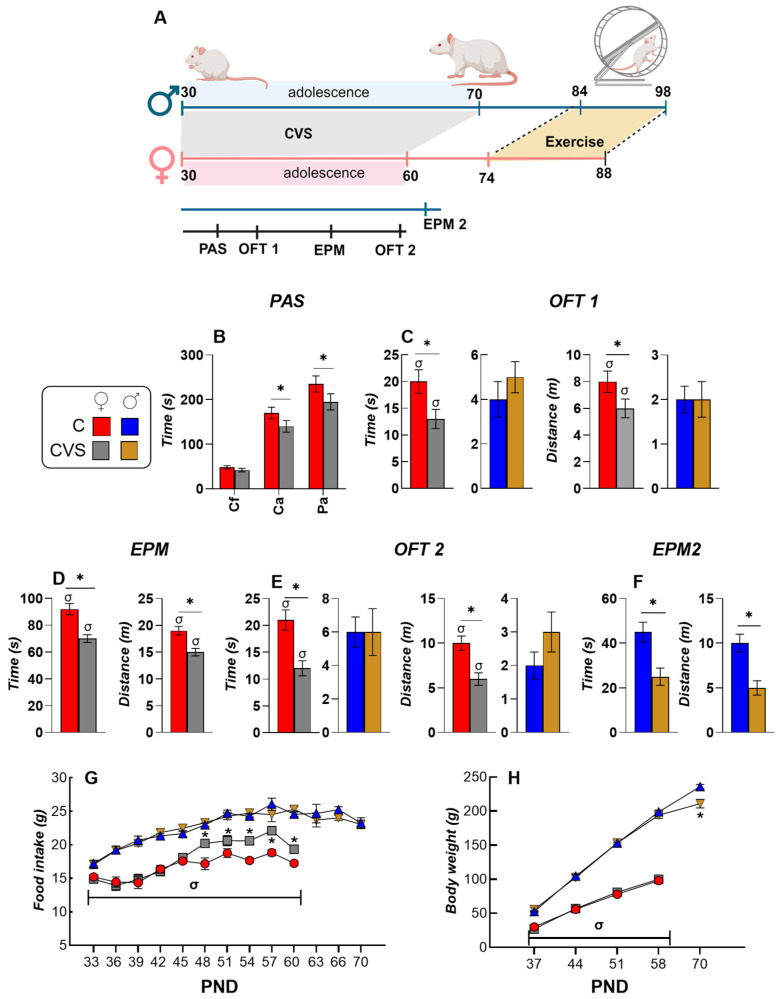
Chronic variable stress (CVS) during adolescence affects anxiety-like behavior, food intake, and body weight in female rats more than in males. (**A**) Male and female rats underwent the CVS paradigm during adolescence (PND30–70 for males and PND30–60 for females). Behavioral tests were conducted throughout the CVS procedure to assess changes during this period. Exercise was started 39 days after the onset of puberty (PND45 for males and PND35 for females). The figure was created using BioRender. (**B**) Spontaneous locomotor activity was assessed using a photobeam activity system (PAS) on PND37 in female rats. Measurements included fine and ambulatory movements in the center of the cage (Cf and Ca) and ambulatory activity in the periphery (Pa). The time spent in the center and the distance traveled in the open field test (OFT) were recorded at PND44 (OFT1; (**C**)) and PND51 (OFT2; (**E**)) for both sexes. The time and distance traveled in the open arms of the elevated plus maze test (EPM) were evaluated at PND58 in females (**D**) and PND65 in males (EPM2; (**F**)). Food intake (**G**) and body weight changes (**H**) were monitored throughout the CVS procedure during adolescence in male and female rats. The results are expressed as mean ± S.E.M. and analyzed by two-way ANOVA for each PND, followed by Tukey’s post hoc test. * *p* < 0.05 vs. C group; σ * *p* < 0.05 vs. males of the same group. C: control group; CVS: chronic variable stress group; PND: postnatal day.

Behavioral tests were conducted throughout the CVS protocol to assess treatment effects (Figure 1A). At PND 37, after seven days of CVS, females showed reduced ambulatory activity in the center and periphery compared to controls (C) (Figure 1B), while males did not. At PND 44, in the open field test (OFT), CVS females spent less time and traveled shorter distances in the center than C females; males did not show center-related differences, although their total distance was lower than that of C females (Figure 1C). Anxiety-like behavior was more evident in the EPM. At PND 51, CVS females spent less time and covered less distance in open arms than C females (Figure 1D), while male groups showed no differences. OFT was repeated at PND 58, again revealing reduced center time and distance in CVS females only (Figure 1E). At PND 65, CVS males exhibited an increased anxiety-like behavior in EPM compared to controls (Figure 1F).

Overall, behavioral evaluations during CVS aligned with previous reports of increased female sensitivity to adolescent stress, reflected in increased emotional reactivity in OFT and EPM compared to males [52,53,56].

Food intake was higher in males than in females (Figure 1G), but relative food intake (RFI, per body weight) was greater in females [42] (Supplementary Table S3). CVS females increased their food intake during the final two weeks of treatment (Figure 1G) without changes in body weight (BW) (Figure 1H). CVS males did not show any change in food intake (Figure 1G), but had reduced BW at PND 70 (Figure 1H), resulting in higher RFI and lower food efficiency (FE) than controls [56] (Supplementary Table S3).

### 2.2. Long-Term Effects of CVS on Voluntary Exercise

**Figure 2:**
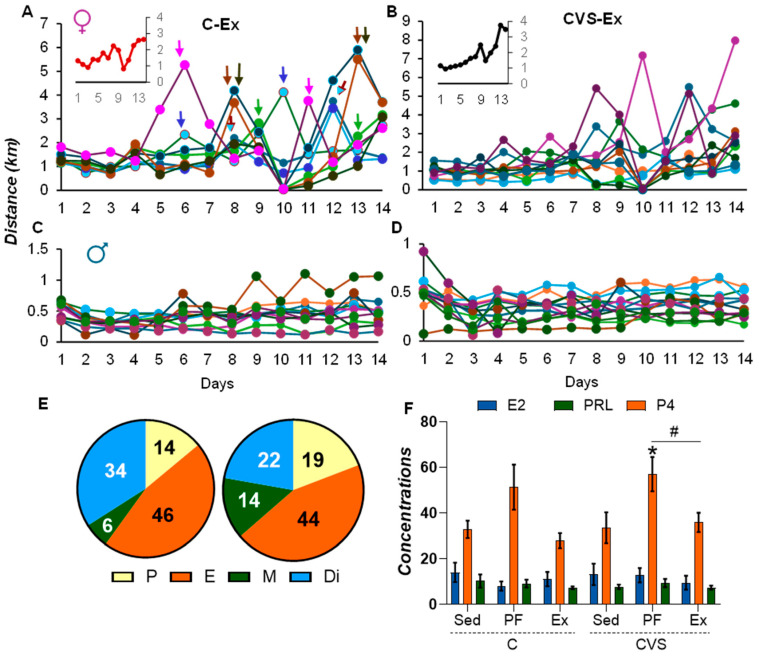
CVS does not affect the activity patterns of cycling female rats or their hormonal profiles. The daily exercise patterns of female control (**A**) and CVS (**B**) reveal periodic peaks aligned with the estrous cycle (C-Ex, indicated by arrows), contrasting with the flat activity trend observed in male control (**C**) and CVS (**D**) rats. (**E**) The distribution of the estrous cycle phases of the control and stressed groups was similar (the figure shows all female rats). (**F**) Serum concentrations of estradiol (E2, pg/mL), prolactin (PRL, ng/mL), and progesterone (P4, ng/mL) in sedentary (Sed), pair-fed (PF), or exercised (Ex) female rats under control or CVS conditions. Stress had a significant effect on serum progesterone levels (*p* = 0.001). The results are expressed as mean ± S.E.M. and analyzed by two-way ANOVA followed by Tukey’s post hoc test. *: *p* < 0.05 vs. C-SED; #: *p* < 0.05 vs. CVS-Ex. P, proestrus; E, estrus; M, metestrus; Di, diestrus. In panels **A**–**D**, different colors are used to represent individual animals.

Daily running plots revealed peaks in C-Ex females, spaced 4–5 days apart, consistent with estrous cycling [57] (Figure 2A), contrasting with the stable pattern in C-Ex males (Figure 2C). Similar trends were observed in the CVS-Ex groups (Figure 2B,D). Females progressively increased their daily running distance (inserts, Figure 2A,B) and exhibited a total distance five times greater than males (female: C-Ex: 23.27 km; CVS-Ex: 25.74 km; male: C-Ex: 5.89 km; CVS-Ex: 4.99 km), which is consistent with previous reports [35,42,47] and greater locomotion in OFT (Figure 1C,E).

To avoid stress, vaginal smears were taken post-decapitation to assess the cycle phase. Estrous phases were evenly distributed between groups, with minor changes in the diestrus/metaestrus percentage (Figure 2E). Serum estradiol and prolactin concentrations were comparable between the groups; however, serum progesterone concentration was elevated in CVS pair-fed females (Figure 2F). These findings support previous evidence that random cycling does not significantly impact exercise performance [58].

### 2.3. Voluntary Exercise Reduces Food Intake, Body Weight, and Fat Mass

**Figure 3:**
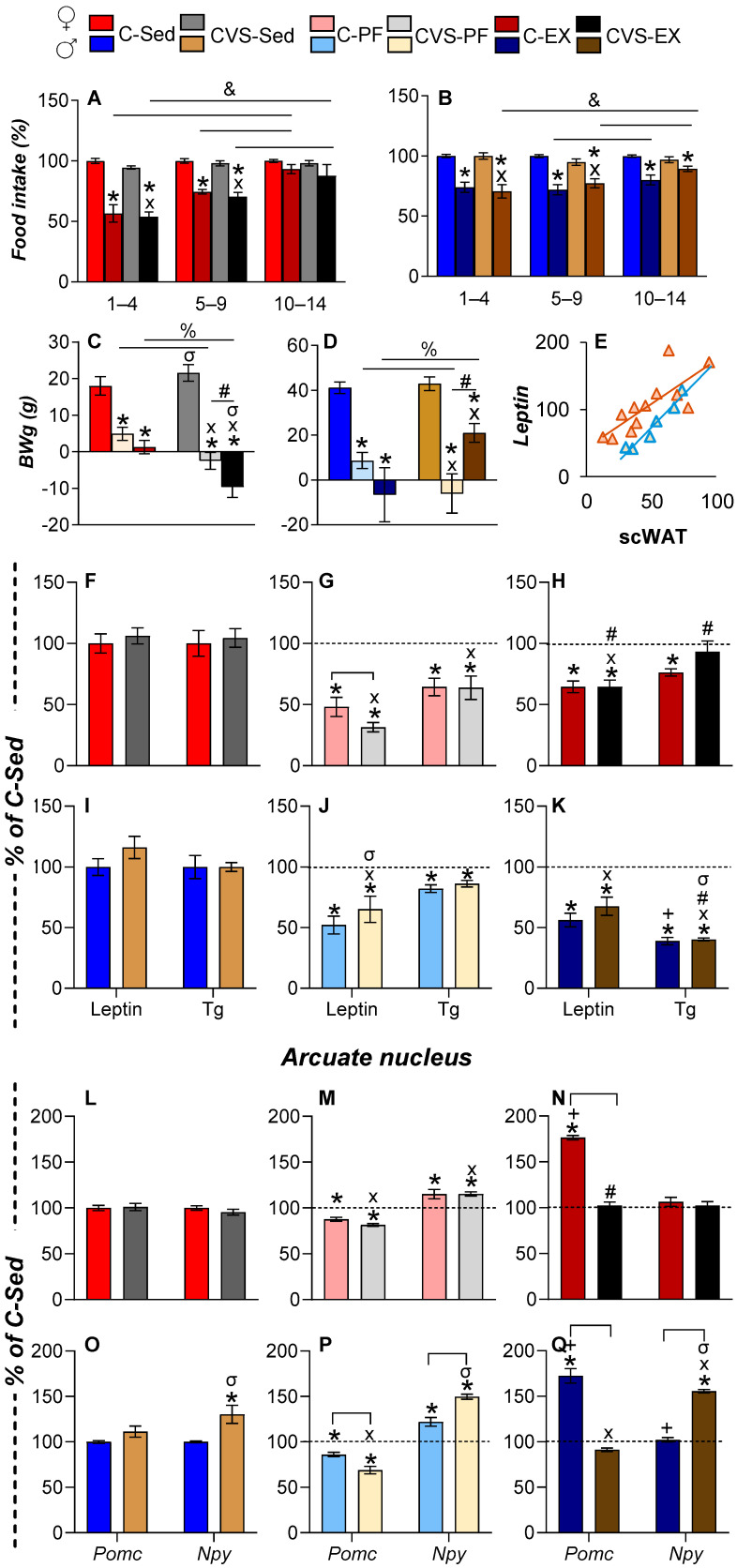
CVS affects the voluntary exercise response of ponderal variables, metabolic hormones, and mediobasal hypothalamic neuropeptides in a sex-dependent manner. Female rats began voluntary exercise at PND74 and males at PND84 (considering the adulthood of each sex). Average food consumption during the 14-day exercise protocol in females (**A**) and males (**B**). Body weight gain (BWg) of females (**C**) and males (**D**). Correlation between interscapular subcutaneous (sc) WAT weight and serum leptin concentration in PF groups of female (pink triangles) and male (grey-blue triangles) rats (**E**). Serum leptin and triglyceride (Tg) concentrations in female (**F**–**H**) and male (**I**–**K**) rats. Pro-opiomelanocortin (*Pomc*) and neuropeptide Y (*Npy*) mRNA levels in the mediobasal hypothalamus (MBH) of female (**L**–**N**) and male (**O**–**Q**) rats. Results are presented as the mean ± SEM, expressed as a percentage of the C-Sed group, and were analyzed using a three-way ANOVA followed by Tukey’s post hoc test. The main effect of exercise was found for weights of adipose tissue (*p* < 0.0001) and for serum leptin and triglyceride concentrations (*p* < 0.0001). A significant interaction was observed between exercise and sex for triglyceride levels (*p* < 0.0001). *Pomc* and *Npy* were significantly affected by activity and stress (*p* < 0.000). *The expression of Npy* was influenced by the main effect of sex (*p* < 0.0001). &: *p* < 0.05 vs. different day periods within the same group (panel A,B); %: *p* < 0.05 vs. CVS-PF or CVS-Ex (panel C,D); *: *p* < 0.05 vs. C-Sed; X: *p* < 0.05 vs. CVS-Sed; #: *p* < 0.05 vs. CVS-PF; +: vs. C-PF; σ: *p* < 0.05 vs. females of the same group. C: control; CVS: chronic variable stress; Sed: sedentary; PF: pair-fed; Ex: exercise.

As reported in [35,42,49], two weeks of WR reduced food intake took place in both sexes, especially during the first five days, with gradual normalization in females (Figure 3A,B). C-Sed females consumed less food than males, and WR reduced intake by 20–28% (Table 1), which is less than the 40% threshold considered mild and beneficial [59].

Body weight gain (BWg) in sedentary rats was 7% to 10% of the initial BW (Table 1). C-PF rats gained less weight than C-Sed rats, while CVS-PF and C-Ex rats showed minimal gain, except for CVS-Ex males, whose BWg exceeded that of CVS-PF (Figure 3C,D, Table 1). Fat mass did not reflect the BW trends: the weight of visceral pgWAT decreased only in C-PF and CVS-PF females, while WR reduced pgWAT weight and subcutaneous WAT (scWAT) weight in all groups (Table 1), which is consistent with the utilization of the depot depending on sex and task [60].

### 2.4. Long-Term Effects of CVS on Serum Energy Markers and ARC Expression Under Mild Food Restriction or Exercise

WAT secretes various hormones, such as leptin, which signal the energy state of the organism. As expected, serum leptin concentration was correlated with scWAT mass (Figure 3E) [61]. Leptin concentration decreased with food restriction and exercise in females (Figure 3F–H), more markedly in CVS-PF than in C-PF (Figure 3G), probably due to stress-induced suppression [62]. This reduction was partially reversed in CVS-Ex (Figure 3H), supporting the role of exercise in buffering stress [63]. In males, leptin concentration decreased similarly between the PF and Ex groups (Figure 3I–K), despite the unchanged WAT mass in PF (Table 1), corresponding to the influence of the overall energy state on leptin release [61].

Serum triglycerides (Tg) and free fatty acids serve as key energy substrates. Tg levels generally reflect WAT mass, increasing postprandially and decreasing with chronic stress, cold, or exercise [48,49,64,65]. Tg decreased in the C-PF, CVS-PF, and C-Ex groups, but not in the CVS-Ex group (Figure 3F–H); in males, Tg dropped modestly in C-PF and markedly in both Ex groups (Figure 3I–K), despite less fat loss compared to females. These patterns suggest sex-specific Tg clearance dynamics and source variability (e.g., postprandial vs. WAT lipolysis), consistent with human and murine data [64,65].

ARC POMC and NPY neurons regulate energy homeostasis by sensing circulating leptin, which stimulates POMC and inhibits NPY neuronal activity. In PF rats, food restriction reduced *Pomc* and increased *Npy* expression in both sexes (Figure 3L–Q), which is consistent with previous reports [61]. Exercise markedly increased *Pomc* expression in C-Ex rats of both sexes (Figure 3N,Q), despite reduced serum leptin levels (Figure 3H,K), supporting the role of POMC neurons in the promotion of physical activity and the existence of stimulus-specific subpopulations [11,66]. However, this exercise-induced upregulation of *Pomc* expression was abolished in CVS-Ex rats (Figure 3N,Q), and *Pomc* expression was further reduced in CVS-PF, especially in males (Figure 3M,P).

Although leptin levels decreased similarly in the PF and Ex groups, *Npy* expression diverged: it increased in PF but not in exercised rats (Figure 3M,P vs. Figure 3N,Q), suggesting differential activation of the subtypes of ARC NPY neurons, some of which were responsive to food restriction and others were inhibited by exercise [67,68,69]. In particular, CVS-Sed and CVS-Ex males showed elevated expression of *Npy* (Figure 3O,Q), which is consistent with chronic stress models, while females responded like controls: increased expression of MBH *Npy* in CVS-PF but no change in CVS-Ex (Figure 3M,P, vs. Figure 3N,Q). This sex-specific stress response may reflect the stimulatory effects of corticosterone and testosterone on *Npy* transcription in males, counteracted by estradiol in females [70,71].

### 2.5. CVS Alters HPA Axis Responses to Mild Food Restriction and Exercise

**Figure 4:**
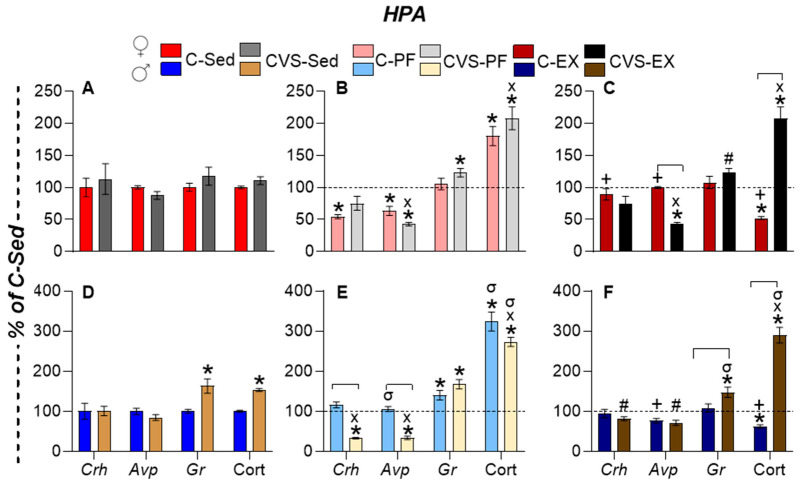
Impact of CVS and voluntary exercise on activity of the hypothalamic–pituitary–adrenal (HPA) axis. Serum corticosterone (Cort) concentration of *Crh*, *Avp*, and *Gr* mRNA levels in the medial caudal paraventricular nucleus (PVN) of female (**A**–**C**) and male (**D**–**F**) rats. The results are presented as the mean ± SEM, expressed as a percentage of the C-Sed group, and were analyzed using a three-way ANOVA followed by Tukey’s post hoc test. Exercise was a significant factor in *Crh* (*p* = 0.004) and *Avp* (*p* < 0.0001) expression levels. Stress also affected *Avp expression* (*p* < 0.0001) and *Gr* expression (*p* = 0.0002), while sex had a significant effect on *Gr* alone (*p* < 0.0001). Analysis of corticosterone (Cort) levels revealed a significant interaction between activity, stress, and sex (*p* = 0.0001), and each factor independently showed a significant main effect (*p* < 0.0001). *: *p* < 0.05 vs. C-Sed; X: *p* < 0.05 vs. CVS-Sed; #: *p* < 0.05 vs. CVS-PF; +: vs. C-PF; σ: *p* < 0.05 vs. females of the same group. C: control; CVS: chronic variable stress; Sed: sedentary; PF: pair-fed; Ex: exercise.

To assess the long-term effects of adolescent CVS on the HPA axis, we examined basal and challenge-induced activity in adulthood. Females had a higher basal Cort concentration than males (C-Sed: 275 ± 26 ng/mL vs. 49 ± 3 ng/mL) [35,72], but CVS alone did not alter the expression of PVN *Crh*, *Avp, and Gr* or serum Cort concentration in C-Sed females (Figure 4A). Calorie restriction reduced PVN *Crh* and *Avp* expression in both the C-PF and CVS-PF groups, while PVN *Gr* expression increased in CVS-PF (Figure 4B). Due to the variability of data, the reduction in *Crh* expression in CVS-PF did not reach significance. Exercise normalized PVN *Crh* and *Avp* expression and serum Cort concentration in C-Ex, but did not do so in CVS-Ex, in which *Avp* expression remained reduced (Figure 4C).

Males showed increased PVN *Gr* expression and serum Cort concentration in CVS-Sed (Figure 4D). The expression of PVN *Crh* and *Avp* decreased in CVS-PF males (Figure 4E), consistent with previous findings in calorie-restricted rats exposed to stress [67,73]. All PF groups showed elevated PVN *Gr* expression and serum Cort concentration, as reported [74] (Figure 4F). Exercise reduced PVN *Gr* expression and serum Cort concentration in C-Ex males (Figure 4F) below C-Sed levels (Figure 4D), despite reports of an increase in serum Cort concentration induced by WR [42,75], reinforcing its stress-buffering effect [47]; this normalization did not occur in CVS-Ex males, whose Cort concentration remained elevated, as in CVS-PF (Figure 4E).

### 2.6. Long-Term Effects of CVS on HPT Axis Activity Under Mild Food Restriction and Exercise

Given our previous findings that voluntary exercise-induced mild food restriction inhibits HPT axis activity in males [49], we included a pair-fed (PF) and a naive group to disentangle the neuroendocrine effects of exercise from those of reduced caloric intake in stressed males and females. This design also allowed us to assess whether central changes in HPT axis regulation translate into peripheral hormone levels and target organ responses.

At the hypothalamic level, hypophysiotropic TRH neurons’ activity and TRH output are modulated by two key enzymes expressed in tanycytes within the MBH: DIO2, which generates T3 for negative feedback on PVN *Trh* transcription [76], and the ectoenzyme degrading thyrotropin-releasing hormone (TRH-DE), which inactivates the TRH released at the median eminence [77]. Since both enzymes are sensitive to nutritional status, studied mainly in fasting [19,76,77,78], we included their evaluation as part of the elements that participate in the setting of HPT axis activity.

#### 2.6.1. Females

**Figure 5:**
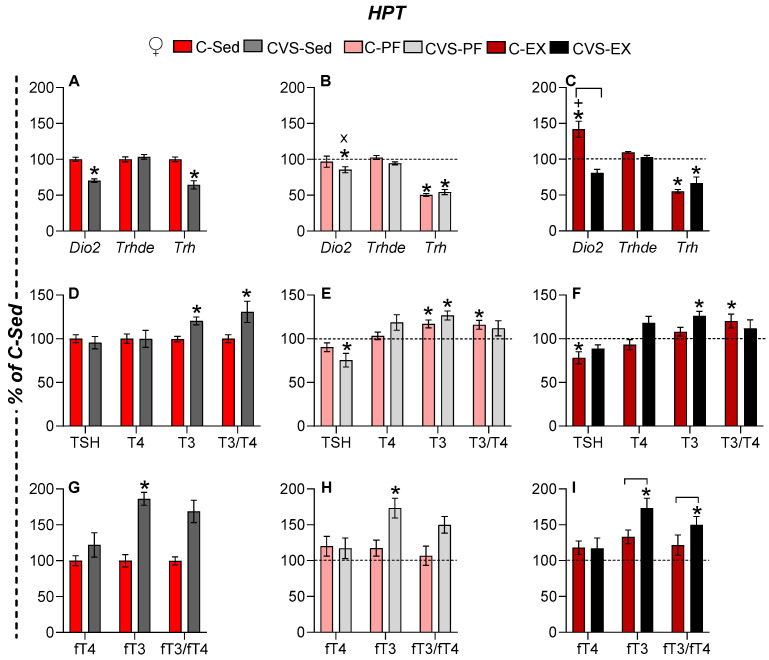
CVS affects the basal activity of the hypothalamus–pituitary–thyroid (HPT) axis and its response to voluntary exercise in female rats. (**A**–**C**) Levels of mRNA of deiodinase type 2 (*Dio2*) and TRH-degrading ectoenzyme (*Trhde*) in the mediobasal hypothalamus (MBH) and of *Trh* in the medial-caudal paraventricular nucleus (PVN). (**D**–**F**) Serum concentrations of TSH, total T4, and T3 and T3/T4 ratios. (**G**–**I**) Serum concentrations of free T4 and T3 (fT4 and fT3) and the fT3/fT4 ratio. The results are presented as the mean ± SEM, expressed as a percentage of the C-Sed group, and were analyzed using a three-way ANOVA followed by Tukey’s post hoc test. Exercise and stress affected the levels of MBH *Dio2* and MBH and PVN *Trh* mRNA, respectively (*p* < 0.0001). ANOVA indicated a significant main effect of sex on the expression of MBH *Trhde* and PVN *Trh* (*p* = 0.001). A significant main effect of activity was also found for serum TSH (*p* < 0.0001) and T4 (*p* = 0.03) concentrations. The effect of stress was significant for T4 (*p* = 0.03), T3 (*p* = 0.0006), and fT3 (*p* < 0.0001), whereas sex influenced the concentrations of T3, fT4, and fT3 (*p* < 0.001). *: *p* < 0.05 vs. C-Sed; X: *p* < 0.05 vs. CVS-Sed; +: vs. C-PF. C: control; CVS: chronic variable stress; Sed: sedentary; PF: pair-fed; Ex: exercise.

MBH *Dio2* expression was reduced in all groups of chronic stress (CVS-Sed), but was increased by exercise (C-Ex females) (Figure 5A–C). The expression of MBH *Trhde* did not change (Figure 5A–C). The expression of PVN *Trh* was suppressed by chronic stress (CVS-Sed), mild food restriction (C-PF, CVS-PF), and exercise (C-Ex, CVS-Ex) (Figure 5A,B). Although the literature reports diverge on the impact of caloric restriction between sexes [59,79], our conditions revealed a reduction in serum TSH concentration only in CVS-PF (Figure 5E), which is consistent with the findings in young female rats kept in a stressful situation as individual housing and subjected to 33% food restriction for 3 weeks [80]. Serum TSH levels also decreased in C-Ex but not in CVS-Ex (Figure 5F), suggesting that additional regulatory mechanisms are at play beyond the control of PVN *Trh* expression. Serum T4 levels remained stable between the groups, while T3 concentrations increased in the C-PF group and all CVS groups (Figure 5D–F). Consequently, the T3/T4 ratio increased in C-PF, C-Ex, and CVS-Sed, but not in CVS-PF or CVS-Ex (Figure 5D–F). Free thyroid hormones were evaluated only in Experiment 2 (*n* = 5–6/group). Serum fT3 concentration was elevated in all CVS groups compared to sedentary controls (Figure 5G–I), indicating a primary effect of chronic stress. The fT3/fT4 ratio, reflecting the peripheral conversion of T4 to T3, followed the trends of serum fT3 concentration but reached statistical significance only in CVS-Ex (Figure 5I).

#### 2.6.2. Males

**Figure 6:**
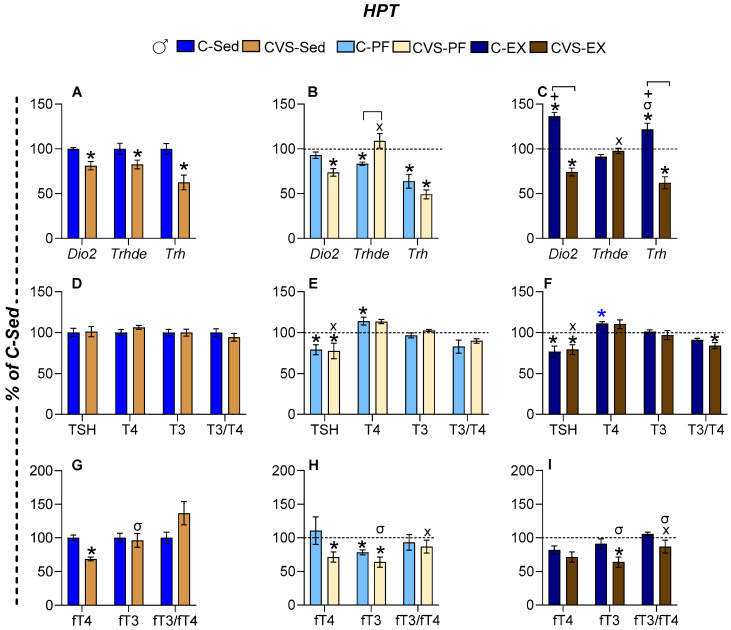
Impact of CVS on the basal activity of the hypothalamus–pituitary–thyroid axis (HPT) and its response to voluntary exercise in male rats. (**A**–**C**) Levels of mRNA expression of the deiodinase type 2 (*Dio2*) and TRH degrading enzyme (*Trhde*) in the mediobasal hypothalamus (MBH) and *Trh* in the medial-caudal paraventricular nucleus (PVN). (**D**–**F**) Serum concentrations of TSH, total T4, and T3 and T3/T4 ratios. (**G**–**I**) Serum concentrations of free T4 and T3 (fT4 and fT3) and the fT3/fT4 ratio. Results are presented as the mean ± SEM, expressed as a percentage of the C-Sed group, and were analyzed using a three-way ANOVA followed by Tukey’s post hoc test. *: *p* < 0.05 vs. C-Sed; X: *p* < 0.05 vs. CVS-Sed; +: vs. C-PF; σ: *p* < 0.05 vs. females of the same group. C: control; CVS: chronic variable stress; Sed: sedentary; PF: pair-fed; Ex: exercise.

In males, the expression of MBH *Dio2* was not affected by 14 days of 25% caloric restriction (C-PF), in contrast to the reported increase in male rats subjected to 40% food restriction for 25 days [78]; it increased in C-Ex [42,49], but it was reduced in all CVS groups (Figure 6A–C). The expression of MBH *Trhde* decreased in C-PF while it increased in CVS-PF and CVS-Ex compared to CVS-Sed, reaching levels comparable to those of C-Sed (Figure 6A–C). PVN *Trh* mRNA levels increased in C-Ex but decreased in the C-PF and all CVS groups, as previously published in exercised and PF male rats [35,42,49] (Figure 6A–C). Serum TSH concentration decreased in response to both caloric restriction and exercise in controls (C-PF, C-Ex) and similarly in all stressed groups (CVS-Sed, CVS-PF, CVS-Ex) (Figure 6D–F). Serum T4 concentration tended to increase, reaching significance in C-PF and near significance in C-Ex (*p* = 0.05); no changes were observed in CVS groups (Figure 6D–F). The T3/T4 ratio decreased under stress in the CVS-Ex group (Figure 6F). The serum fT4 concentration decreased in CVS-Sed and CVS-PF, while CVS-Ex showed a nonsignificant upward trend (Figure 6G–I). The serum fT3 concentration was reduced in the C-PF, CVS-PF, and CVS-Ex groups. Consequently, the fT3/fT4 ratio was altered only in stressed groups, with differences between CVS-Sed and both CVS-PF and CVS-Ex, although with high variability (Figure 6G–I).

The results show similar hypothalamic responses of male and female rats to CVS, with decreased expression of MBH *Dio2* and *Trh* in sedentary or pair-fed groups, but expression was stimulated by exercise; however, in C-Ex females, *Trh* values remained as low as in C-PF, as previously published [42,49]; calorie restriction reduced MBH *Trh* expression in both sexes. CVS inhibited the activity of the HPT axis in male rats in all groups, decreased MBH *Trh* expression, as well as serum concentrations of TSH and fT4, a result that differs from females, who showed no change in serum fT4 and instead had increased T3 and fT3 concentrations in response to CVS.

### 2.7. Long-Term Effects of CVS on Gene Expression in Target Organs

#### 2.7.1. Skeletal Muscle (SKM)

**Figure 7:**
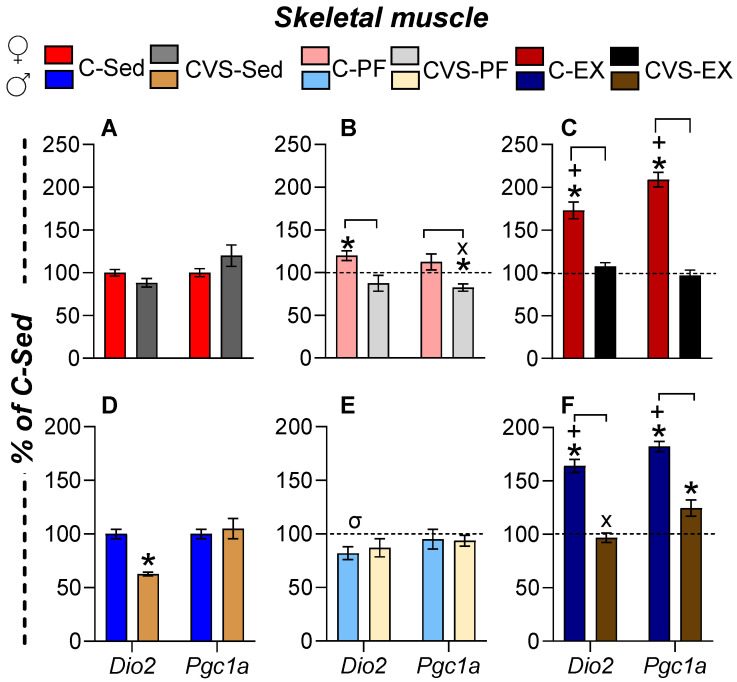
CVS alters the response to voluntary exercise in the expression of genes that regulate the effects of thyroid hormones in skeletal muscles in a sex-dependent manner. mRNA levels of deiodinase type 2 (*Dio2*) and peroxisome proliferator-activated receptor γ coactivator 1α (*Pgc1a*) in the skeletal muscle (gastrocnemius muscle) of female (**A**–**C**) and male (**D**–**F**) rats. Results are presented as the mean ± SEM, expressed as a percentage of the C-Sed group, and were analyzed using a three-way ANOVA followed by Tukey’s post hoc test. The expression of *Dio2* and *Pgc1a* was significantly affected by exercise (*p* < 0.0001) and stress (*p* < 0.0001). Furthermore, a main effect of sex (*p* = 0.0003) on *Dio2* expression was observed. *: *p* < 0.05 vs. C-Sed; X: *p* < 0.05 vs. CVS-Sed; +: vs. C-PF; σ: *p* < 0.05 vs. females of the same group. C: control; CVS: chronic variable stress; Sed: sedentary; PF: pair-fed; Ex: exercise.

Exercise begins with SKM contraction, triggering metabolic adaptations and signaling cascades that influence peripheral tissues [10,81,82]. T3 regulates muscle metabolism via PGC1α, which promotes mitochondrial biogenesis, fatty acid oxidation, and fiber-type switching [22,23,83,84]. DIO2, modulated by stress and exercise [25,85,86], also contributes to local T3 production; fatty acid oxidation and tissue adaptations continue during early post-exercise recovery (up to 4h) [48]. In C-Ex female rats, SKM *Dio2* and *Pgc1a* expression increased (Figure 7C), while pair-feeding had minimal impact, except for a modest increase in *Dio2* mRNA levels in C-PF; CVS hampered these increments (Figure 7A,B). In males, the expression of SKM *Dio2* was also increased in C-Ex, together with that of *Pgc1a,* and decreased in CVS-Ex, although the expression of *Pgc1a* remained partially elevated (Figure 7D–F).

#### 2.7.2. White Adipose Tissue

Lipolysis in WAT is driven by β3-adrenergic receptor (Adrb3), which also stimulates the expression of *Dio2*, the proliferator-activated receptor γ (*Pparg*), and hormone-sensitive lipase (HSL, *Lipe*) through cAMP-PKA signaling [87,88,89,90]. PPARγ is a transcription factor that acts as a lipid sensor and is involved in many aspects of fatty acid metabolism and glucose homeostasis [90], exercise-induced browning of iWAT, and glucose homeostasis in visceral WAT as pgWAT [88]. HSL is the rate-limiting enzyme in triglyceride hydrolysis [89], and its expression is increased by glucocorticoids, exercise, and calorie restriction [89,91,92,93]. We evaluated gene expression in subcutaneous iWAT, a tissue that undergoes several adaptations to exercise, and in visceral WAT (pgWAT), which provides fuel in negative energy situations and whose utilization differs depending on task and sex [60,94].

**Figure 8:**
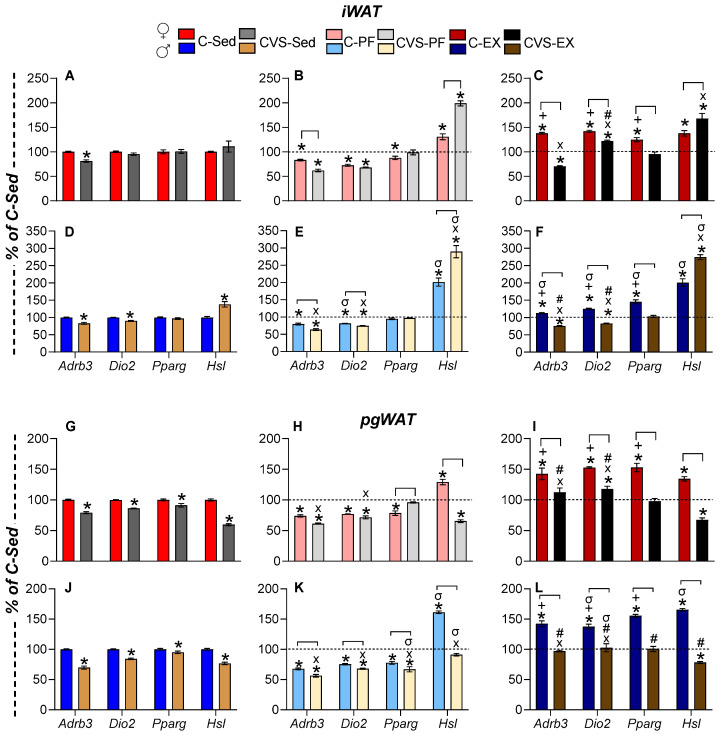
Sex differences in the expression of genes that regulate lipid metabolism in inguinal (iWAT) and perigonadal (pgWAT) white adipose tissues. mRNA levels of the adrenergic beta 3 receptor (*Adrb3*), deiodinase type 2 (*Dio2*), peroxisome proliferator-activated receptor gamma (*Pparg*), and hormone-sensitive lipase (*Hsl; Lipe*) in iWAT of female (**A**–**C**) and male (**D**–**F**) rats. mRNA levels of *Adrb3*, *Dio2*, *Pparg*, and *Hsl* (*Lipe*) in pgWAT of female (**G**–**I**) and male (**J**–**L**) rats. The results are presented as the mean ± SEM, expressed as a percentage of the C-Sed group, and analyzed using a three-way ANOVA followed by Tukey’s post hoc test. Exercise (*p* < 0.0001) and stress (*p* < 0.0001) had significant effects on *Adrb3*, *Dio2*, *Pparg,* and *Hsl* expression in both fat depots. The main effect of sex was also significant for almost all genes, including *Adrb3*, *Dio2*, and *Hsl*, in both tissues (*p* < 0.001). The exception was *Pparg*, which was significantly affected by sex only in iWAT (*p* = 0.001) but not in pgWAT. *: *p* < 0.05 vs. C-Sed; X: *p* < 0.05 vs. CVS-Sed; #: *p* < 0.05 vs. CVS-PF; +: vs. C-PF; σ: *p* < 0.05 vs. females of the same group. C: control; CVS: chronic variable stress; Sed: sedentary; PF: pair-fed; Ex: exercise.

The response of both depots was similar: chronic stress reduced *Adrb3* expression in pgWAT and iWAT depots of CVS-Sed rats of both sexes (Figure 8A,D,G,J), probably due to glucocorticoid-mediated inhibition [95,96]. Food restriction also suppressed the expression of *Adrb3* and *Dio2,* more in CVS-PF than in C-PF (Figure 8B,E,H,K). The expression of *Pparg* decreased in iWAT of C-PF females and in pgWAT of female C-PF, and only in male CVS-PF (Figure 8K), in agreement with reports on male rats exposed to chronic unpredictable stress as adults [88,97].

Exercise increased the expression of *Adrb3*, *Dio2*, and *Pparg* in both depots (Figure 8C,F,I,L), albeit to a lower degree in male iWAT (Figure 8F). Stress prevented these responses, particularly in iWAT, where CVS-Ex levels were lower than those of the C-Sed group, except for female iWAT *Dio2*, where CVS-Ex levels exceeded CVS-Sed but remained below C-Ex (Figure 8C,F,I,L).

Unlike other genes, *Hsl* (*Lipe*) expression in inguinal white adipose tissue (iWAT) increased in response to stress and food restriction. This upregulation was observed in sedentary males exposed to chronic variable stress (CVS-Sed; Figure 8D), as well as in pair-fed (C-PF) and chronically exercised (C-Ex) rats, with higher levels in males, even though females ran longer distances (Figure 8B,C,E,F).

#### 2.7.3. Brown Adipose Tissue (BAT)

BAT is a highly metabolically active adipose tissue activated by cold exposure, where norepinephrine and T3 act in synergistic ways that enhance thermogenesis through the production of heat by mitochondria due to the activation of the uncoupling protein 1 (UCP1) [98]. The involvement of BAT in exercise has been questioned due to conflicting results, but its participation in lipid and glucose homeostasis, as well as its activation during exercise, is increasingly recognized [59,91,99].

**Figure 9:**
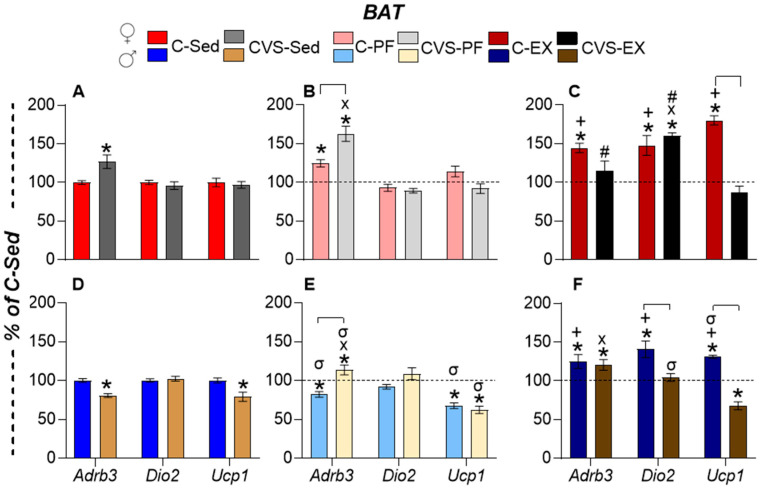
CVS alters the effects of voluntary exercise on thermogenic gene expression in brown adipose tissue (BAT) in a sex-dimorphic manner. mRNA levels of the adrenergic beta 3 receptor (*Adrb3*), deiodinase type 2 (*Dio2*), and uncoupling protein 1 (*Ucp1*) in brown adipose tissue from female (**A**–**C**) and male (**D**–**F**) rats. Results are presented as the mean ± SEM, expressed as a percentage of the C-Sed group, and were analyzed using a three-way ANOVA followed by Tukey’s post hoc test. Exercise influenced the expression of *Adrb3*, *Dio2,* and *Ucp1* (*p* < 0.0001 for all). The main effect of sex was also significant for *Adrb3* and *Ucp1* expression (*p* < 0.0001). Finally, a significant effect of stress was observed only for *Ucp1* (*p* < 0.0001). *: *p* < 0.05 vs. C-Sed; X: *p* < 0.05 vs. CVS-Sed; #: *p* < 0.05 vs. CVS-PF; +: vs. C-PF; σ: *p* < 0.05 vs. females of the same group. C: control; CVS: chronic variable stress; Sed: sedentary; PF: pair-fed; Ex: exercise.

The BAT responses differed according to sex. Females had a higher expression of *Adrb3* in CVS-Sed, C-PF, CVS-PF, or C-Ex than in C-Sed (Figure 9A–C). The expression of *Dio2* and *Ucp1* was increased only by exercise, *Dio2* in C-Ex and CVS-Ex, and *Ucp1* only in C-Ex (Figure 9C). Males, in contrast, had decreased expression of *Adrb3* in CVS-Sed and C-PF, but increased expression in CVS-PF (Figure 9D,E); *Ucp1* expression was reduced in CVS-Sed, C-PF, and CVS-PF (Figure 9D,E).

The response to exercise was attenuated in males compared to females, with lower stimulation of all gene expression. *Adrb3* expression was higher in C-Ex and CVS-Ex than in C-Sed, while *Dio2* expression was blunted and *Ucp1* expression inhibited (Figure 9F).

The results summarized in Table 2 reveal the effects of differential stress on adult responses to energy challenges, such as mild calorie restriction (CR) and voluntary exercise. Exercise induced the expression of *Dio2* in all tissues studied, together with the transcription factors *Pgc1a* or *Pparg* in muscle or WAT, and *Ucp1* in BAT. Mild food restriction exerted an inhibitory effect on the expression of most markers. Chronic stress during the adolescent period suppressed most of these changes.

### 2.8. Correlation Analyses

**Figure 10:**
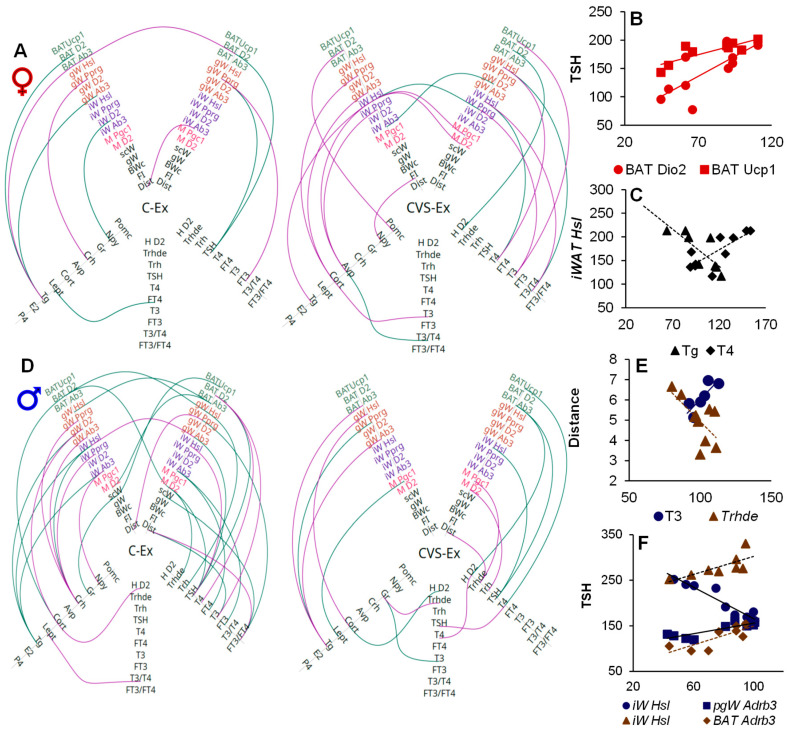
Hive plot of the correlations between gene expression in the hypothalamus, peripheral tissues, and serum hormones of control and CVS-exercised rats. Hive plot diagrams illustrate significant (*p* < 0.05) Pearson correlations between gene expression in the hypothalamus and peripheral tissues and circulating hormone concentrations in exercised female (**A**) and male (**D**) rats. Green lines denote positive correlations, and the purple lines denote negative correlations. (**B**) In C-Ex females, mRNA levels of BAT *Dio2* and *Ucp1* were positively correlated with serum TSH concentrations. (**C**) In CVS-Ex females, iWAT *Hsl* (*Lipe*) mRNA levels showed a negative correlation with serum triacylglycerol (Tg) and a positive correlation with serum T4 concentrations. (**E**) In C-Ex males, the running distance was positively correlated with serum T3 levels, while in CVS-Ex males, it was negatively correlated with *Trhde* expression. (**F**) Serum TSH concentrations correlated with iWAT *Hsl* (*Lipe*) expression in both C-Ex and CVS-Ex males, and with *Adrb3* expression in pgWAT of C-Ex males and BAT of CVS-Ex males. H, hypothalamus; gW, perigonadal WAT; iW, inguinal WAT; M, skeletal muscle; scW, interscapular subcutaneous WAT; FI, food intake; BWc, body weight change; Dist, total distance traveled (exercise performed). Hive plots created with software: https://codeberg.org/rgarcia-herrera/neuro_endocrine_correlation/ (accessed on 19 September 2025).

We have previously shown that exercise-induced changes in PVN *Trh* expression or serum T4 or T3 concentration are linearly correlated with the distance traveled by male or female rats [35,42,49]. We performed FDR-adjusted *p*-value analyses to diminish the risk of false positives. Variations in serum T3 concentrations were significantly associated with distance. These analyses revealed several changes in gene expression or serum hormone concentrations that were proportional to variations in several of the variables measured and between different tissues. Although they do not represent a causal relationship, various correlations reflected known physiological functions, which supports the reliability of the results. Representing the correlations among the different variables in the hive plots allowed us to recognize many associations in exercised rats between components of the HPT axis and target molecules in peripheral tissues (Figure 10). Importantly, the hive plots revealed correlations that support the concerted responses of various tissues to energy demands and open new questions for future research.

Exercise-induced associations between various elements of the HPT axis (Figure 10). For example, in females and males, PVN *Trh* expression was negatively correlated with ARC *Npy* expression, which is consistent with the inhibitory action of NPY on *Trh* expression [78]. Serum TSH concentration was associated with many other variables, supporting its role in various aspects of metabolism through its receptor present in many tissues; for example, TSH stimulates *Dio2* in cultured brown adipocytes [100,101]. A positive effect of T4 on Adrb3 expression has been reported in [21]. The positive correlation between T4 concentration and *Hsl* expression coincides with the lipolytic actions of T4 [22]. Stress hindered most of the C-Ex correlations, but generated new ones, which were more negative than those in C-Ex (Figure 10 and Supplementary Figures S3 and S4). Many more variables were correlated in C-Ex males than in C-Ex females. Compared to the C-Ex group, fewer associations were found in pair-fed groups and even less in sedentary rats, many of them different from those registered in exercised groups (Supplementary Figures S3 and S4). The hive plots illustrated various hubs in which one variable was associated with several others; for example, the correlations between the expression of ARC *Npy*, or the serum concentration of Cort, and several elements of the HPT axis in C-PF males (Supplementary Figure S4) were consistent with their interaction under conditions of energy deficit and with the role of NPY neurons in the regulation of energy expenditure [78,102]. Multiple associations are justified by the effects of TH and TSH, including the presence of TSH receptors in several tissues [100,101,103]. The correlation of PVN *Trh* expression with SKM and BAT gene expression is supported by the effect of TRHergic neurons of PVN on sympathetic neurons that contact these tissues [104].

## 3. Discussion

Although exercise is widely recognized for mitigating stress-related disturbances, most studies emphasize previous or concurrent exercise relative to subsequent responses to a variety of psychological or social stressors [4,5,6,40]. In contrast, the impact of prior chronic stress on subsequent exercise outcomes remains less explored [41], and, in particular, the metabolic and neuroendocrine consequences. Understanding the mechanisms involved is hampered by the multiple interactions between neuroendocrine axes and autonomic circuits. Although chronic stress can alter neuroendocrine axes and contribute to disease vulnerability, its long-term impact can remain undetectable if basal hormonal activity appears to be unaffected by early life stressors, as occurs with the HPT axis [14,15,21,78]. However, such latent alterations can become evident under physiological challenges. In this study, which was carried out on chronically stressed Wistar rats, we demonstrate that while stress-induced changes in the classical markers of HPT axis activity were modest and variable, the expression of genes responsive to thyroid hormones in key metabolic tissues was significantly altered during exercise. These findings suggest that developmental stress can prime target tissues for altered thyroid hormone signaling [105], with functional consequences emerging only under metabolic demand.

Experiments with adolescent rats submitted to CVS and tested during the procedure confirmed that females were susceptible to stress effects from early puberty, while males showed anxious behavior throughout adolescence, possibly reflecting a near-adult stage [4,5,52,53,54,56,106,107]. Validation of the paradigm through the evaluation of the activity of the HPA axis revealed persistent effects of CVS applied during adolescence in both sexes. Although CVS increased anxiety-like behavior in females during adolescence, alterations in the basal activity of the HPA axis were observed in adulthood only in males, with elevated serum corticosterone concentration and expression of PVN GR and ARC *Npy* [52,53].

**Figure 11:**
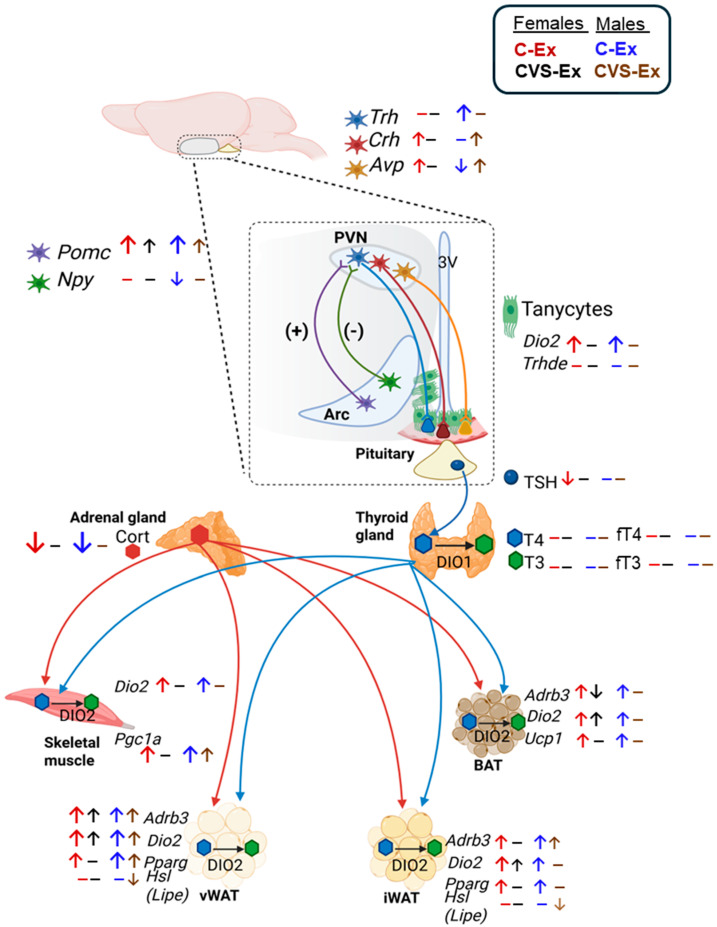
Effects of exercise on HPT and HPA axes, and target tissues of female and male control and chronic variable stressed rats (CVS) compared to the pair-fed group. The diagram summarizes the long-term sex-dependent effects of adolescent chronic variable stress (CVS) and of adult voluntary exercise (Ex) on key components of the hypothalamic–pituitary–thyroid (HPT) and adrenal (HPA) axes and on thyroid hormone-responsive genes in peripheral tissues of rats. The groups are represented by different colors for males and females; upward arrows (↑) indicate an increase, downward arrows (↓) indicate a decrease, arrow sizes illustrate the degree of change, and dashes (−) indicate no significant change, relative to the C-PF or CVS-PF group. C: control; CVS: chronic variable stress; PF: pair-fed; Ex: exercise. Image created using biorender.com.

As with many animal experiments, the design of controls is difficult, the typical “control” being sedentary rats left in animal facilities without any disturbance other than cage change and weight measurements every week. Compared to this control, we observed that HPT axis activity was inhibited in the group pair-fed to the exercise group, probably due to the mild, forced calorie restriction in conjunction with isolation at night, suggesting an adaptive mechanism [59]. Pair-fed controls have the same calorie intake as the exercised rats; in previous publications, we used PF as controls [35,42]. However, as illustrated in Figure 11, whose arrows show differences between exercised and pair-fed rats, some changes are overlooked; for example, the effects of CVS on MBH *Npy* expression in males, or serum T3 concentration in females. The voluntary reduction in food intake of exercised rats could be due to the release of free fatty acids, which remain elevated hours after exercise, serve as nutrients [48], and could be detected in the brain, although direct evidence for this is lacking. Rats were decapitated after two weeks of exercise, 3 h after light on, and returned to the cage with their mate; this corresponds to the post-exercise recovery period, during fluctuating sleep and feeding bouts, when measurements likely represent the cumulative effect of two weeks of mild diet or of exercise training, and not an acute response.

During exercise, energy use is enhanced by the work performed in muscle contraction and cardiorespiratory effort, thus activating the use of energy reserves through lipolysis. Consistent with previous reports [42,49], exercised rats consumed 24 to 28% less food than controls, which is considered a mild calorie restriction proposed to extend lifespan [59], but it also triggered an elevated serum corticosterone concentration [74,108]. Females ran more and consumed less food than males, explaining the greater loss of pgWAT weight in pair-fed females; although the most expected metabolic changes induced by decreased food intake appeared in both sexes in different proportions, the reductions in serum leptin concentration and ARC *Pomc* expression, along with increases in ARC *Npy* expression, were similar in pair-fed rats of both sexes [61,62,73]. Exercise stimulated MBH *Pomc* expression similarly in unstressed males and females, despite the longer running distance of females than males; expression changes are consistent with exercise-induced increases in ARC POMC neuron firing and suppression of NPY neuron activity [69], even though leptin concentration decreased during exercise. Previous CVS exposure attenuated this response, and MBH *Pomc* expression did not correlate with distance run, likely due to neuronal functional heterogeneity in receptors and projection targets [11,66,69]. Chronic restraint stress reduces excitatory input to ARC POMC neurons, decreasing their responsiveness [109].

Voluntary exercise decreased serum Cort concentration in both sexes but had no effect on other elements of HPA activity [42,75]; in contrast, CR inhibited central HPA activity (reduced PVN *Crh* and *Avp* expression) in C-PF and CVS-PF females and only in CVS-PF males [67,73]. PF-induced inhibition of PVN *Crh* and *Avp* expression does not appear to be due to direct feedback effects, as serum Cort concentration increased similarly in all groups, but increased PVN *Gr* expression followed a different pattern. Even if serum Cort concentration increased, central HPA activity was neither altered in C-PF males, nor PVN *Crh* expression in CVS-Sed males. Increased MBH *Npy* expression in CVS males indicates stimulation of NPY/AgRP neuron activity; because NPY/AgRP terminals in the median eminence release AgRP that increases CRH secretion without affecting PVN *Crh* expression [110], it could contribute to sex differences.

Different types of stress [27,28,55] and energy deficit conditions inhibit HPT axis activity at multiple levels [59,78,79,80]. Elements of the hypothalamic–pituitary–thyroid (HPT) axis exhibited subtle but significant changes in response to exercise or food restriction, consistent with previous reports [42,49]. In contrast, the response to chronic variable stress (CVS) revealed long-term alterations in adult rats, including reduced *Trh* expression in the paraventricular nucleus (PVN). This finding differs from previous studies showing no change when CVS is applied to male rats or mice of both sexes sacrificed the day following the stress protocol [39,111]. *Dio2* inhibition differed from the reported increased expression after chronic corticosterone administration [112] or mild food restriction [78], the latter being attributed to a decrease in serum leptin and an increase in serum Cort concentrations. Fasting-induced tertiary hypothyroidism [34,76,79,113] has been attributed to increased *Dio2* expression in tanycytes, which enhances local T3 production and, later, suppresses *Trh* transcription [76,113]. However, while pair-fed (C-PF) rats of both sexes exhibited low serum leptin and elevated serum corticosterone concentrations, and CVS-Sed rats showed elevated serum corticosterone concentration alone, MBH *Dio2* expression remained unchanged in C-PF animals, but was reduced in both CVS-Sed and CVS-PF groups. Therefore, the mechanisms underlying the long-term inhibitory effects of CVS on MBH *Dio2* expression remain to be elucidated [19]. The expression of MBH *Trhde,* which parallels the expression of *Dio2* in fasted male rats due to the stimulating effects of thyroid hormones on *Trhde* [34,77,78], also decreased in CVS-Sed and C-PF males but normalized in the CVS-PF and CVS-Ex groups, supporting additional regulatory factors beyond DIO2-generated T3. The sexually dimorphic effects of CVS were most evident in the serum concentrations of peripheral circulating hormones; in all CVS groups of males, the serum concentration of fT4 was reduced, while CVS females showed an increase in serum T3 concentration, and an even higher concentration of fT3. The reductions in serum fT4 concentration in male rats, together with those in serum TSH concentration and PVN *Trh* expression, align with tertiary hypothyroidism induced by energy deficit [35,42,49,59,80], which was present in the PF and CVS-Ex groups as stress prevented the stimulatory effect of exercise. CVS-induced increases in female serum T3 concentration, particularly fT3, suggest additional players that will be discussed below.

Skeletal muscle is a primary target for thyroid hormones that are critical for muscle function and maintenance, as well as for exercise performance [10,24,25,83,84,85,86]. Among the tissues analyzed, the expression of *Dio2* in the skeletal muscle showed the highest response to exercise in both sexes (Supplementary Figure S2), most likely leading to an increased concentration of T3 in the gastrocnemius muscle, as recently shown in male rats after repeated mild treadmill running [114]. T3 regulates the expression of *Pgc1a*, and its dependence on exercise-induced *Dio2* stimulation is demonstrated after treatment with iopanoic acid. Furthermore, in SKM-Dio2-KO mice [25], the blunting of *Dio2* response by CVS explains the decreased upregulation of *Pgc1a* that can impair the utilization of energy substrates and maintenance of muscle fiber. However, CVS suppressed *Pgc1a* expression only partially, supporting that additional elements regulate this transcription factor [115].

WAT depots supply energy substrates in a sex-specific manner [60,116,117]. Exercise induces subcutaneous WAT adaptations, including sympathetic innervation, mitochondrial biogenesis, and increased expression of genes involved in lipid and glucose homeostasis [116,118]. Visceral WAT lipolysis is also induced by exercise and negative energy balance conditions; although this depot contains fewer amounts of adrenergic receptors, it regulates glucose homeostasis and inflammatory tone via cytokines [9,119]. We measured the parameters stimulated by exercise or TH in WAT and were able to reproduce the data (cited individually in the Section 2): exercise improved, while CR reduced the expression of *Adrb3*, *Dio2*, and *Pparg*. Comparing the data between WATs revealed that the sex differences detected in the magnitude of responses of each depot were smaller for *the* expression of *Adrb3* and *Dio2* in the iWAT of exercised males than of females, probably due to the higher running activity of the latter. Only in the pgWAT of CVS-PF males, the expression of *Pparg* was lower than in C-PF, and these results aligned with stress-induced glucose intolerance [88,97].

The expression of *Hsl* in response to CVS, caloric restriction (CR), or exercise varied by adipose depot and sex. It was significantly elevated in both the inguinal and perigonadal depots of chronically exercised (C-Ex) males, with a more modest response in females. Unlike most evaluated genes, whose expression was suppressed by chronic stress, *Hsl* was upregulated in iWAT but inhibited in pgWAT of CVS-PF animals of both sexes. This divergent pattern underscores depot-specific regulation and suggests differential sensitivity to stress and intervention. The literature on CR-induced modulation of HSL remains inconclusive [89,92,93], reflecting its regulation by multiple signaling pathways. Adrenergic agonists rapidly enhance HSL enzymatic activity without altering mRNA levels, whereas dexamethasone increases *Hsl* mRNA expression over threefold [92]. These findings imply that additional factors may contribute to the suppression of *Hsl* expression in pgWAT of groups exhibiting elevated corticosterone levels (CVS-PF and CVS-Ex). Chronic stress is well recognized to promote visceral fat accumulation in both animals and humans, but the underlying mechanisms remain incompletely understood. Inhibition of *Hsl* expression may represent a contributing factor to this phenotype and deserves further investigation.

Brown adipose tissue (BAT), whose role in exercise was once questioned, is now recognized as strongly activated by physical activity [91,120], as evidenced by the increased expression of *Adrb3*, *Dio2*, and *Ucp1*. *Ucp1* induction in male mice is associated with muscle-derived factors induced by exercise, such as IL-16 [121]. Unlike other tissues that showed inhibited expression of several markers by CR and the suppressive effect of stress on exercise-induced stimulation in the expression of most of the genes analyzed, including BAT *Dio2* in males, the expression of *Dio2* in female BAT of CVS-Ex was stimulated to the same magnitude as that of C-Ex, and that of CVS-PF was even higher than C-PF. Differences between male and female BAT metabolism have been recognized; under ad libitum feeding conditions, female rats present higher thermogenic activity, which is reduced to a greater extent under 40% calorie restriction than in males, by decreasing *Ucp1* expression [122]. These differences have been proposed to be due to the crosstalk between glucocorticoids and sex hormones even at the level of transcriptional receptors [123]. However, a local effect of this crosstalk during exercise is unlikely, since *Ucp1* expression was inhibited in females as in males, suggesting the involvement of other factors. Beyond energy expenditure, CVS-stimulated increase in BAT *Dio2* may contribute to elevated circulating T3 concentration, as has been proposed in other paradigms [124,125]. Additional mechanisms may contribute to the observed effects, including inhibition of T3 inactivation pathways such as DIO3- or DIO1-mediated deiodination, or inhibition of enzymes involved in thyroid hormone (TH) clearance. In this regard, in vitro studies indicate that glucocorticoids reduce DIO3 activity [126], but they concurrently upregulate hepatic enzymes responsible for TH clearance [127]. These seemingly opposing actions underscore the complexity of the regulatory network, and the precise mechanisms involved remain to be elucidated. Additionally, the increase in fT3 concentration was higher than that of T3 concentration in CVS groups, suggesting the participation of additional regulatory players, such as thyroid hormone-binding proteins. In other stress paradigms, including maternal separation [34], we have not detected such an increase in T3 concentration in stressed males [27,37,39,128] or in stressed females, except after 2 weeks of repeated intermittent restraint [34,35,42,79]. These data cast doubt on the general effect of glucocorticoids but support particular long-term allostatic changes in females stressed during adolescence. The exclusive response of female BAT suggests that CVS during adolescence altered circuits that can interact with those involved in HPT responses.

The results obtained in Wistar rats offer the advantage of controlling multiple experimental variables, providing a robust framework for identifying the neural and endocrine mechanisms underlying stress-related metabolic adaptations. These findings may guide future research toward clinically relevant models aimed at understanding human pathophysiology. In particular, elevated serum concentrations of triiodothyronine (T3) and thyroxine-binding globulin have been reported in post-war veterans with post-traumatic stress disorder (PTSD) [129], and increased levels of T3 and free T3 (fT3) have also been observed in other psychiatric conditions, including bipolar I disorder [130,131]. Such parallels underscore the translational potential of these preclinical models in elucidating thyroid axis dysregulation in stress-related disorders. Comparative studies are required to characterize time-dependent endocrine and behavioral adaptations to prolonged exercise that contribute to improved resilience to stress [40,47,106,132,133].

The pattern of peripheral organ responses was remarkably consistent, and the observed correlations between tissues suggest a coordinated, system-wide adaptation. In both sexes, exercise increased the expression of ARC *Pomc* alongside the adrenergic receptor β3 in adipose tissues and *Dio2* in tanycytes and peripheral organs. This was accompanied by an increase in the expression of key metabolic regulators, including *Pgc1a*, *Pparg*, and *Ucp1*. On the contrary, chronic variable stress (CVS) broadly attenuated these responses, with the notable exception of brown adipose tissue (BAT) in females. These findings raise compelling questions about the central mechanisms that mediate these effects. Given that CVS induces plastic changes in neural circuits that govern stress, exercise, and cognition, a plausible site of action may involve afferent pathways to the hypothalamus that mature during adolescence [11,40,47,66]. The disruption of POMC neuron signaling by chronic stress [109] could be a key mechanism, as these neurons exert multisynaptic control over skeletal muscle, WAT, and BAT activity [11,66,134].

In conclusion, chronic stress during adolescence produced sex-dimorphic effects on the metabolic and endocrine responses to exercise in adulthood. Our findings demonstrate that exposure of adolescent rats to chronic variable stress leads to persistent activation of HPA axis and inhibition of the hypothalamic–pituitary–thyroid (HPT) axis in males, while females exhibited elevated serum concentrations of T3 and free T3 (fT3). CVS disrupted the exercise-induced expression of genes that regulate HPT axis activity and peripheral metabolic tissues in a sex-dependent manner. Exercise did not reverse the long-term effects of adolescent stress, suggesting that short-term physical activity may be insufficient to counteract CVS-induced neuroendocrine reprogramming, particularly in females. These results underscore the importance of developmental timing and sex as critical variables in stress physiology and highlight the need for extended exercise paradigms to fully assess the resilience or vulnerability of neuroendocrine circuits shaped by early life adversity.

## 4. Materials and Methods

### 4.1. Animals and Experimental Groups

Wistar rats were bred and raised in the Institute’s animal pathogen-free installation, with a 12-h light/dark cycle (lights at 7:00 h), independent filtered air, and positive pressure in each room. The rat colony is renewed every five years with 10 nonrelated male and 15 female Wistar rats from Charles River Laboratories (Wilmington, MA, USA). Only two people took care of the animal husbandry, maintaining a strict crossbreeding registry to avoid family intercrossing [39]. Rats were kept with food (Teklad 2018SX, Envigo, Denver, PA, USA) and purified water (in glass bottles to limit endocrine disruptors) ad libitum. All procedures followed the Guide for the care and use of laboratory animals [135], the Mexican norm NOM-062-ZOO-1999, and the approval of the Institute Bioethics Committee (No. 374), taking care to keep stress at a minimum [37,39].

Two independent experiments were performed on males and females. For each experiment, 12 virgin female rats (3 months old) were mated with unrelated males, the day of birth was registered, and the litter was culled to 10 pups/mother; at weaning (PND 21), the pups of each mother were placed by sex in a cage. Each experiment required 6 groups of 5–6 rats each; 3 groups were submitted to CVS, and the others were controls; groups were formed at PND 30, placing one pup of each mother in a different cage, ending with 2 pups/cage using 3 cages/group without siblings; 9 cages were used as control and the other nine for CVS.

Because the period of adolescence extends over puberty, that is restricted to hormonal and physical changes, the period of adolescence was chosen according to [56], from PND 31–60 for females and PND 31–70 for males, to guarantee the inclusion of the sensible periods considering the existing controversies regarding the end of adolescence in rats [5,50,51,106] (Figure 1A). Behavioral tests were conducted throughout the CVS procedure to assess the state of the animals during treatment.

Eighteen rats were subjected to the CVS paradigm daily throughout adolescence, with a different stressor applied each day during the light period. Female rats were exposed for 30 days and males for 40 days, corresponding to the sex-specific adolescent periods defined in [51]. The CVS protocol consisted of a series of unpredictable stressors adapted from [136] with a few modifications: (1) 3 h tilt, (2) 3 h white noise, (3) 3 h strobe light, (4) overnight social isolation, (5) 24 h continuous light, (6) 12 h fasting, (7) 15 min elevated platform, (8) 1 h restraint, (9) 1 h exposure to a cold room (4 °C), and (10) behavioral testing (PAS, OFT, and EPM). After completing the CVS protocol, the animals underwent a 2-week recovery period before beginning the voluntary exercise regimen in adulthood. The detailed CVS schedule is presented in Supplementary Table S1. To prevent stress transmission, CVS rats were housed in a separate room from control (C) and pair-fed (PF) rats, thus avoiding noise generated by cage transfers and by the activity of stressed animals.

### 4.2. Behavioral Tests

The rats were subjected to behavioral tests during the light period, beginning at 10:00 h. Spontaneous locomotor activity at PND 37 was evaluated by placing rats individually for 5 min in a standard cage with a photobeam activity system (PAS-Home Cage, San Diego Instruments Co., San Diego, CA, USA) connected to a computer that registers central and peripheral activities, ambulatory and fine movements, and rearing. Photobeams were placed around the cage, and the program distinguished fine movements from locomotion.

At PND 44 and 58, rats were subjected to the OFT. They were placed for 5 min and left to explore freely in an enclosed square arena of 100 × 100 × 60 cm made of opaque black acrylic (San Diego Instruments Co.), under dim instead of bright light to distinguish small changes [129]. The total distance traveled (measure of total locomotion), the distance and time spent in the center of the area, and the number of entries to the center were registered and analyzed with the SMART 2.5 software (Panlab, Barcelona, Spain). A reduced time and distance spent in the center indicate anxiety behavior (inferred by the avoidance of crossing the center of the field).

At PND 51, both male and female rats, at PND 65, only male rats were subjected to the EPM [129] (San Diego Instruments Co.). The rats were placed for 5 min in the center of the EPM; the time spent in the open or closed arms, and the distance traveled were recorded and analyzed with the software SMART 2.5 (Panlab).

### 4.3. Voluntary Exercise

Two weeks after the end of adolescence and CVS or C treatment, rats were exposed to voluntary wheel running (WR), at PND 74 for females and PND 84 for males [50]. The control and CVS animals were divided into 3 groups (5–6 rats/group), avoiding siblings within the groups: sedentary (Sed) fed ad libitum, pair-fed (PF) to match the food intake of exercised rats, and exercised (Ex) [42,49]. C-Sed and CVS-Sed rats were left undisturbed in a different room from the Ex and PF groups. As rodents are nocturnal and run most of the time during the dark period [46], C-Ex and CVS-Ex rats were placed individually, only during the dark period (at light change, from 19:00 to 7:00 h) for 14 days, in a cage with a running wheel (AccuScan Instruments Inc., Columbus, OH, USA) with fresh food and water; at 7:00 h, the animals were returned to their cage mate with food and water. Food intake was monitored after each cage change (at 7:00 h and 19:00 h), and distance was recorded at 7:00 h. The average amount of food consumed by the Ex rats in each period was provided to the PF rats; during the dark period, the control PF rats were placed in individual cages to account for the 12 h stress of isolation [137] that the exercised rats experienced in the cage with the running wheel; during the light period, they were returned to their home cage mate.

### 4.4. Tissue Collection

After 2 weeks of voluntary wheel running (WR), rats were euthanized on day 15, three hours after lights on. Thus, biochemical measurements represent values during the recovery phase from the final exercise bout, with less than 3 h since the cessation of activity. Food intake (FI) and body weight (BW) were recorded on the last day of exercise (females: FI = 20 ± 1 g, BW = 256 ± 14 g; males: FI = 24 ± 1 g, BW = 435 ± 17 g). Euthanasia was performed by decapitation using a sharp guillotine, performed by an experienced technician, starting at 09:00 h. To control for circadian variations in hormone or mRNA levels, animals from different experimental groups were processed in an alternating order.

The trunk blood was collected immediately, serum was separated by centrifugation, aliquoted, and stored at −20 °C. To avoid stress, vaginal smears were obtained directly after decapitation to determine the estrous cycle stage by cytology [138]. The brains were carefully removed, severing the optic nerve prior to extraction to preserve the median eminence, and placed in aluminum foil on dry ice. Peripheral tissues, including the gastrocnemius muscle, WAT, and BAT, were rapidly dissected. Perigonadal (pg), retroperitoneal (r), and interscapular (isc) WAT depots were fresh weighed before freezing, while the iWAT was dissected and frozen without weighing to prevent RNA degradation during prolonged handling. All tissues were wrapped in aluminum foil and stored at −70 °C. Dissections were performed by a team of at least five trained personnel to minimize the interval between euthanasia and tissue preservation.

### 4.5. Brain Dissections

Frozen brains were placed in a cryostat (Thermo Scientific HM525, Waltham, MA, USA) for coronal sectioning; after verifying alignment and recognizing the anterior/median boundary of the PVN in 20 μm slices (toluidine blue staining), three consecutive 200 μm coronal slices were cut, which included the median-caudal PVN (Bregma −1.3 to −1.9 mm) and 5 other slices for the mediobasal hypothalamus (MBH, Bregma −1.92 to −2.92 mm) [139]. Each section was placed horizontally on a glass cover of a Petri dish over dry ice. For the 3 anterior slices, the PVN from each side of the third ventricle was cut with a 0.5 mm internal diameter sample corer (Fine Science Tools, Foster City, CA, USA) and stored in tubes at −70 °C. The following 5 slices included the ARC and the median eminence with the lower third of the 3rd ventricle containing the β2 tanycytes that express *Dio2* and thyrotropin-releasing hormone-degrading ectoenzyme (*Trhde*) [77]; for each slice, a sample corer of 0.5 mm internal diameter was placed at the center and most ventral end of the hypothalamus.

### 4.6. Hormonal Measurements

TSH (NIDDK reagents and protocol, antibody ID AB_2891204) and Cort (Merck-Millipore, antibody ID AB_90543) were quantified by radioimmunoassay and other hormones with ELISA kits following the manufacturer’s instructions, except that for total T3 (cat. IIDE 2021, Diagnóstica International, Zapopan, México) and T4 (cat. IIDE 2022, Diagnóstica International), an aliquot of 25 µL of hypothyroid rat serum was added to the standard curves, as reported in [42]. Free T4 (fT4) (cat. E-EL-0122) and free T3 (fT3) (cat. E-EL-0079) were measured using Elabscience kits only in the sera of rats from experiment 2. Estradiol (cat. 80548), progesterone (cat. 80558), prolactin (cat. 80560), and leptin (cat. 90040) were quantified using Crystal Chem (Elk Grove Village, IL, USA) kits. The samples were measured in duplicate; the mean was taken as n = 1; the intra-assay and inter-assay coefficients of variation were <10%.

### 4.7. RNA Extraction and mRNA Quantification

Total RNA was isolated from PVN, MBH, and gastrocnemius muscle using the acid guanidinium thiocyanate–phenol–chloroform method [140]. For adipose tissues, the modifications included a first centrifugation at 870 g for 10 min and an additional chloroform wash to remove fat. The purity and integrity of the RNA were verified by electrophoresis, and the intensity of the 18S and 28S bands was quantified; samples with a 28/18 ratio of less than 1.5 were discarded. One µg RNA was retrotranscribed (M-MLV, 200 U/µL, Invitrogen, Carlsbad, CA, USA), and cDNA amplified by endpoint PCR for MBH, gastrocnemius muscle, BAT, and WAT using recombinant Taq DNA polymerase (Biotecnologías Moleculares, Mexico City, México), and linearity was verified against internal markers cyclophilin A (*Ppia*) or hypoxanthine phosphoribosyl transferase 1 (*Hprt*) [34,42]. The PVN genes were quantified by qPCR (Sygreen Mix, PCR Biosystems, London, UK) on the RotorGene Q (Qiagen, Hilden, Germany) with an initial denaturing stage at 95 °C for 10 min, followed by 40 cycles at 95 °C for 10 s, annealing temperature of 60 °C for 20 s, and a 72 °C extension period for 20 s. A melting curve analysis was performed to verify that only one PCR product was detected in each sample. The relative expression of the target genes relative to the reference genes was calculated using the Pfaffl method [141]. The expression level of the target genes was normalized against *Hprt* or *Ppia*. The end-point PCR and qPCR primer sequences used are described in Supplementary Table S2.

### 4.8. Statistical Analysis

Two independent experiments were carried out/sex, n = 5–6/group in each experiment; mean of the C-Sed values of each experiment was taken as 100% and the value for each animal calculated as % of the C-Sed mean; the % values of the two experiments were combined, calculated as the mean ± S.E.M. Data were analyzed using multifactorial analysis of variance (ANOVA) to evaluate the effects of stress and sex on the behavioral test performed before the exercise protocol. Subsequently, all data were analyzed to assess the effects of stress, exercise, and sex. After significant main effects or interactions, post hoc pairwise comparisons among the six experimental groups (male and female rats subjected to stress, exercise, or their combination) were made using Tukey’s honestly significant difference (HSD) test. This test was used to control the family-wise error rate (FWER) at an alpha level of 0.05 in all pairwise comparisons. Multiplicity-adjusted *p*-values and 95% confidence intervals are reported for each comparison (Supplementary Table S4A–C). Before analysis, the normality and homogeneity of variance assumptions were assessed; when necessary, data transformations were performed to meet these assumptions. For clarity, results that include group means and confidence intervals are presented in the original percentages or units, while all inferential statistics were calculated on the transformed scale. All statistical analyses were performed with GraphPad Prism version 10.4, and significance was accepted at *p* < 0.05.

Furthermore, Pearson correlation analyses were performed to assess linear associations between the expression levels of genes and hormone concentrations in multiple tissues, using the percentages calculated against the mean of the C-Sed group of each experiment. To account for the risk of false positives arising from multiple hypothesis testing, the resulting *p*-values were adjusted using the False Discovery Rate (FDR) correction according to the Benjamini–Hochberg procedure. Associations were considered statistically significant if the FD-adjusted *p* value was less than 0.05. For visual analysis of correlation networks, we developed hive plots (Figure 10) [142] (created with software: https://codeberg.org/rgarcia-herrera/neuro_endocrine_correlation/ (accessed on 19 September 2025)). Only significant correlations that showed linearity were included, eliminating links between two separate sets of data.

## Figures and Tables

**Table 1:** Summary of food intake (FI), body weight gain (BWg), relative food intake (RFI), and food efficiency (FE), along with the total weight of perigonadal (pg), retroperitoneal (r), and interscapular (isc) white adipose tissue (WAT) depots, after the voluntary exercise period in control (C) and chronic variable stress (CVS) rats of both sexes. Data are presented as mean ± SEM and were analyzed using a three-way ANOVA followed by Tukey’s post hoc test. *: *p* < 0.05 vs. C-Sed; ^#^: *p* < 0.05 vs. C-PF; ^X^: *p* < 0.05 vs. CVS-Sed; ^&^: *p* < 0.05 vs. CVS-PF; ^σ^: *p* < 0.05 vs. male of the same group. Ex, exercise; PF, pair-fed; Sed, sedentary.

	C			CVS		
	Sed	PF	Ex	Sed	PF	Ex
Females						
FI (g/d)	18.7 ± 0.6 ^σ^	14 ± 0.01 * ^σ^	14.3 ± 0.5 * ^σ^	18 ± 0.6 ^σ^	13.4 ± 0.3 * ^X σ^	13.5 ± 0.7 * ^X σ^
RFI (g/day/g BW)	74 ± 4.1 ^σ^	56 ± 1.3 * ^σ^	58 ± 2.3 *	77 ± 2.4 ^σ^	58 ± 1.3 ^X σ^	59 ± 6.9 ^X^
FE (g BWg /100 g food)	5 ± 1.3 ^σ^	2 ± 0.5 *	−1 ± 1.2 *	7 ± 0.8 ^σ^	0.4 ± 1.0 ^Xσ^	−7 ± 2.5 ^X & σ^
%BWch	107 ± 0.9	102 ± 0.6 *	101 ± 0.6 * ^σ^	109 ± 0.9	99 ± 0.8 * ^X^	97 ± 1 * ^X σ^
% pgWAT	100 ± 4	78 ± 6.5 *	61 ± 6 *	115 ± 2	75 ± 4 * ^X σ^	73 ± 7.6 * ^X^
% rWAT	100 ± 3.7	90 ± 8.1	57 ± 7.2 * ^#^	113 ± 13.2	70 ± 5 * ^X^	72 ± 7.4 * ^X^
% iscWAT	100 ± 9.1	106 ± 11.9	74 ± 9.5 * ^#^	117 ± 12.7	96 ± 5.9	74 ± 3.4 * ^X #^
Males						
FI (g/day)	25.3 ± 0.3	19 ± 0.48 *	19 ± 0.9 *	25 ± 0.7	21 ± 0.4 ^X^	20.3 ± 0.7 ^X^
RFI (g/day/g BW)	56 ± 2.0	48 ± 1.6 *	51 ± 3.7	61 ± 1.1	52 ± 1.2 ^X^	51 ± 2.1 ^X^
FE (g BWg /100 g food)	11 ± 0.8	3 ± 2.2 *	3 ± 2.2 *	11 ± 1.3	6 ± 0.9 ^X^	4 ± 1.1 ^X^
%BWch	110 ± 0.7	103 ± 0.9 *	92 ± 2.5 * ^#^	111 ± 0.68	98 ± 2 * ^X^	105 ± 1.2 ^X &^
% pgWAT	100 ± 4.4	84 ± 6.5	76 ± 4.1 *	93 ± 6.8	92 ± 6.9	75 ± 5 * ^X &^
% rWAT	100 ± 5.9	85 ± 6.1	69 ± 4.2 *	87 ± 7.3	87 ± 8.7	69 ± 4.5 *
% iscWAT	100 ± 12.7	88 ± 13.6	69 ± 7.3 *	90 ± 11.6	93 ± 8	68 ± 5.8 * ^X^

**Table 2:** Summary of responses to 14 days of food restriction (pair-fed) or exercise in female or male adult control or previously stressed rats during adolescence (CVS). Arrows indicate significant changes compared to those in the control sedentary group (C-Sed)}, (--) indicates no change compared to control values. The direction of the arrow denotes the nature of the change (↑ increase; ↓ decrease). The number of arrows represent the level of change. Filled cell colors highlight sex-specific effects: either a significant change observed in only one sex or opposite responses between sexes.

	**Females**						**Males**					
	**C**			**CVS**			**C**			**CVS**		
	**Sed**	**PF**	**Ex**	**Sed**	**PF**	**Ex**	**Sed**	**PF**	**Ex**	**Sed**	**PF**	**Ex**
Food intake	--	**↓**	**↓**	--	↓	↓	--	↓	↓	--	↓	↓
ΔBW	--	↓	**↓↓**	--	↓	**↓↓**	--	↓	**↓↓**	--	**↓↓**	↓
pgWAT	--	↓	↓	--	↓	↓	--	--	↓	--	--	↓
rWAT	--	--	↓	--	↓	↓	--	--	↓	--	--	↓
iscWAT	--	--	↓	--	--	↓	--	--	↓	--	--	↓
Leptin	--	↓	↓	--	**↓↓**	↓	--	**↓↓**	↓	--	↓	↓
TG	--	↓	↓	--	**↓↓**	--	--	↓	↓	↓	↓	**↓↓**
*Pomc*	--	↓	**↑**	--	↓	--	--	↓	**↑↑**	--	↓	--
*Npy*	--	↑	--	--	↑	--	--	↑	--	↑	**↑↑**	**↑↑**
*Crh*	--	**↓**	--	--	--	--	--	--	--	--	↓	--
*Avp*	--	**↓**	--	--	**↓↓**	**↓↓**	--	--	--	--	**↓↓**	--
*Gr*	--	--	--	--	↑	--	--	↑	--	**↑↑**	**↑↑**	**↑↑**
Cort	--	**↑↑**	**↓↓**	--	**↑↑**	**↑↑**	--	**↑**	↓	↑	**↑↑**	**↑↑**
MBH *Dio2*	--	--	↑	↓	↓	↓	--	--	↑	↓	↓	↓
*Trhde*	--	--	--	--	--	--	--	↓	--	↓	--	--
*Trh*	--	**↓↓**	**↓↓**	**↓↓**	**↓↓**	**↓↓**	--	↓	↑	↓	**↓↓**	↓
TSH	--	--	↓	--	↓	--	--	↓	↓	--	↓	↓
T4	--	--	--	--	--	--	--	↑	↑	--	--	--
fT4	--	--	--	--	--	--	--	--	--	↓	↓	--
T3	--	↑	--	↑	↑	↑	--	--	--	--	--	--
T3/T4	--	↑	↑	↑	--	--	--	--	--	--	--	↓
fT3	--	--	--	**↑↑**	**↑↑**	**↑↑**	--	↓	--	--	↓	↓
fT3/fT4	--	--	--	--	--	**↑↑**	--	--	--	--	--	--
SM *Dio2*	--	↑	**↑↑**	--	--	--	--	--	**↑↑**	↓	--	--
SM *Pgc1a*	--	--	**↑↑**	--	↓	--	--	--	**↑↑**	--	--	**↑↑**
iW *Adrb3*	--	↓	↑	↓	↓	↓	--	↓	↑	↓	↓	↓
iW *Dio2*	--	↓	↑	--	↓	↑	--	↓	↑	↓	↓	↓
iW *Pparg*	--	↓	↑	--	--	--	--	--	↑	--	--	--
iW *Hsl*	--	↑	↑	--	**↑↑**	**↑↑**	--	**↑↑**	**↑↑**	↑	**↑↑**	**↑↑**
pgW *Adrb3*	--	↓	↑	↓	↓	--	--	↓	↑	↓	**↓↓**	--
pgW *Dio2*	--	↓	**↑↑**	--	↓	↑	--	↓	↑	↓	↓	--
pgW *Pparg*	--	↓	↑	↓	--	--	--	↓	**↑↑**	↓	↓	--
pgW *Hsl*	--	↑	↑	↓	↓	↓	--	**↑↑**	**↑↑**	↓	--	↓
BAT *Adrb3*	--	↑	**↑↑**	↑	**↑**	--	--	↓	↑	↓	↑	↑
BAT *Dio2*	--	--	**↑↑**	--	--	**↑**	--	--	**↑↑**	--	--	--
BAT *Ucp1*	--	--	**↑↑**	--	--	--	--	↓	↑	↓	↓	↓

## Data Availability

The original data presented in the study are available upon reasonable request.

## Supplementary Materials

The following supporting information can be downloaded at: https://www.mdpi.com/article/10.3390/ijms26199425/s1.

## Abbreviations

The following abbreviations are used in this manuscript:

ACTH	Corticotrophin
Adrb3	Adrenergic receptor β3
AgRP	Agouti-related peptide
ARC	Arcuate nucleus
Avp	Arginine vasopressin
BAT	Brown adipose tissue
BW	Body weight
BWg	Body weight gain
C	Control
Cort	Corticosterone
CR	Calorie restriction
CRH	Corticotropin-releasing hormone
CVS	Chronic variable stress
Dio1	Deiodinase 1
Dio2	Deiodinase 2
Dio3	Deiodinase 3
E2	Estradiol
EPM	Elevated plus maze
Ex	Exercised (voluntary wheel running)
FE	Food efficiency
FI	Food intake
fT3	Free T3
fT4	Free T4
Gr	Glucocorticoid receptor
HPA	Hypothalamus–pituitary–adrenal
Hprt	Hypoxanthine phosphoribosyl transferase 1
HPT	Hypothalamic–pituitary–thyroid
HSL (Lipe)	Hormone-sensitive lipase
IL-16	Interleukin-16
iWAT	Inguinal WAT
MBH	Mediobasal hypothalamus
NPY	Neuropeptide Y
OFT	Open Field Test
P4	Progesterone
PAS	Photobeam activity system
PF	Pair-fed
Pgc-1α	Peroxisome proliferator-activated receptor γ coactivator 1α
pgWAT	Perigonadal WAT
PND	Postnatal day
POMC	Pro-opiomelanocortin
Ppia	Cyclophilin A
Pparg	Peroxisome proliferator-activated receptor γ
PRL	Prolactin
PVN	Paraventricular nucleus of the hypothalamus
RFI	Relative food intake
Sed	Sedentary
SKM	Skeletal muscle
T3	Triiodothyronine
T4	Thyroxine
Tg	Triglycerides
TH	Thyroid hormones
TRH	Thyrotropin-releasing hormone
TRH-DE (Trhde)	TRH-degrading ectoenzyme
TSH	Thyrotropin
Ucp1	Uncoupling protein-1
WAT	White adipose tissue
WR	Wheel running
