# Supplementary material for: Long-Term Effects of Stress During Adolescence on the Sex-Dependent Responses of Thyroid Axis and Target Tissues to Exercise in Male and Female Wistar Rats
Source: Int J Mol Sci. 2025 Sep 26;26(19):9425. doi: 10.3390/ijms26199425 (PMC12524718; doi:10.3390/ijms26199425)
Supplement: Supplementary file 1 [file ijms-26-09425-s001.zip › ijms-3793237-supplementary.pdf]

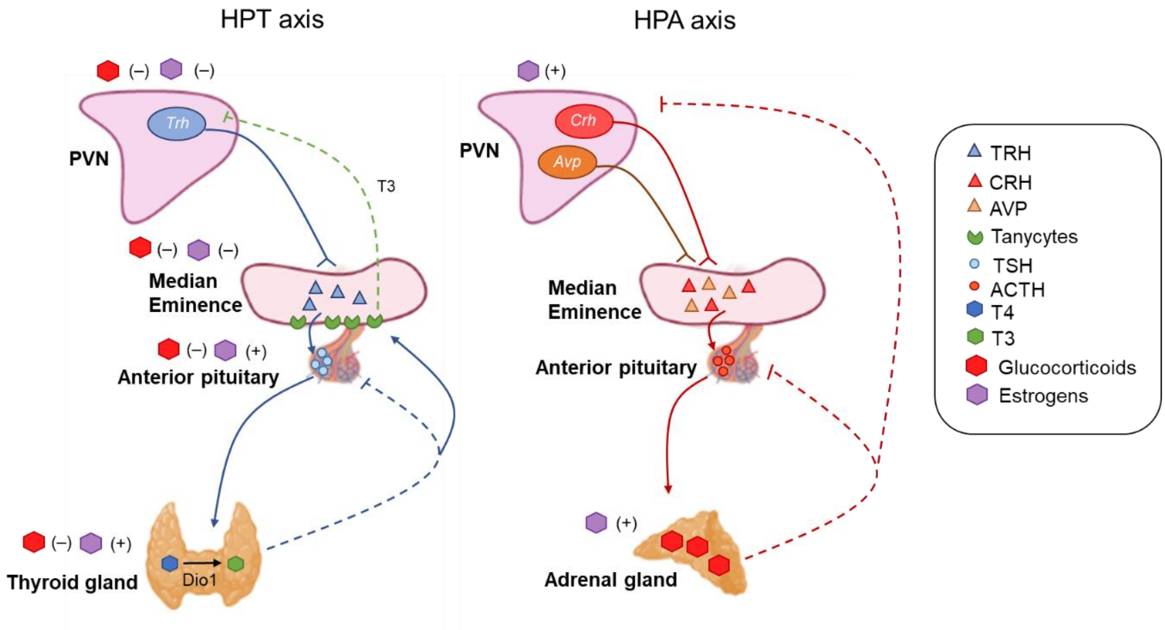

Supplementary Figure S1. Hypothalamic-Pituitary-Thyroid (HPT) and Hypothalamic-Pituitary-Adrenal (HPA) Axes. This diagram illustrates the regulatory mechanisms of two major endocrine axes. The left panel depicts the HPT axis: the paraventricular nucleus (PVN) releases thyrotropin-releasing hormone (TRH), which stimulates the anterior pituitary to release thyroid-stimulating hormone (TSH). TSH acts on the thyroid gland to produce thyroid hormones (T3), with Dio1 facilitating the conversion of T4 to T3. T3 exerts negative feedback on the PVN and the anterior pituitary. The right panel shows the HPA axis: PVN releases corticotropin-releasing hormone (CRH) and arginine vasopressin (AVP), stimulating the anterior pituitary to release adrenocorticotropic hormone (ACTH). ACTH acts on the adrenal gland to produce glucocorticoids (cortisol), which exert negative feedback on the PVN and anterior pituitary. Glucocorticoids and estrogens stimulate (+) or inhibit (-) key components of both axes.

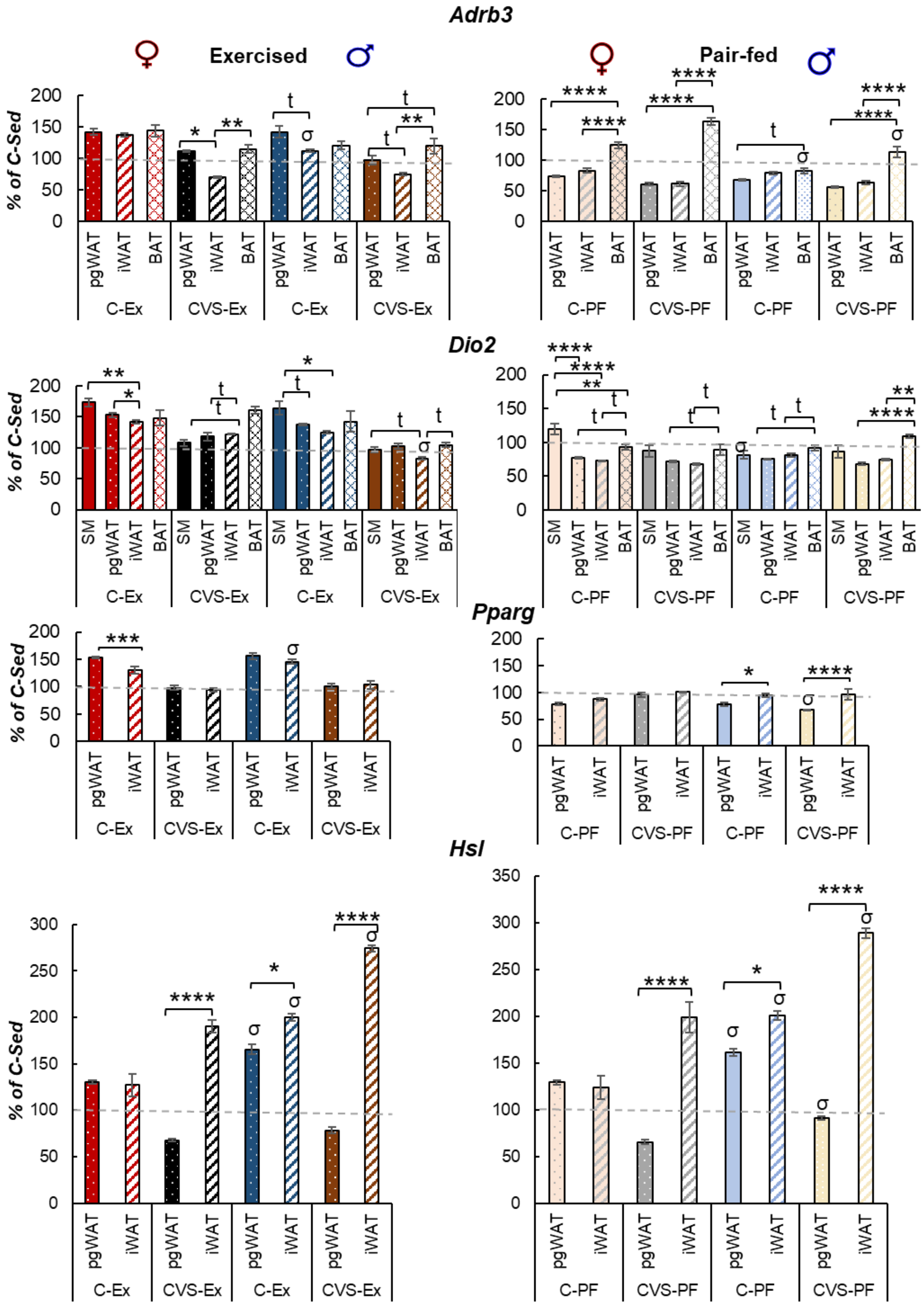

Supplementary Figure S2. Gene expression in adipose tissues and skeletal muscle in response to exercise (Ex) or pair-feeding (PF) in male and female rats. Gene expression levels (as % of C-Sed) of Adrb3, Dio2, Pparg and Hsl (Lipe) in perigonadal (pg) and inguinal (i) white adipose tissue (WAT), brown adipose tissue (BAT) and skeletal muscle (SM). Data are presented as mean ± SEM and were analyzed using two-way ANOVA followed by Tukey's post hoc test.* P<0.05, ** P<0.01, *** P<0.001, **** P<0.0001; σ P<0.05 vs. females in the same group; t significant by t-test. C: control group; CVS: chronic variable stress group.

Females

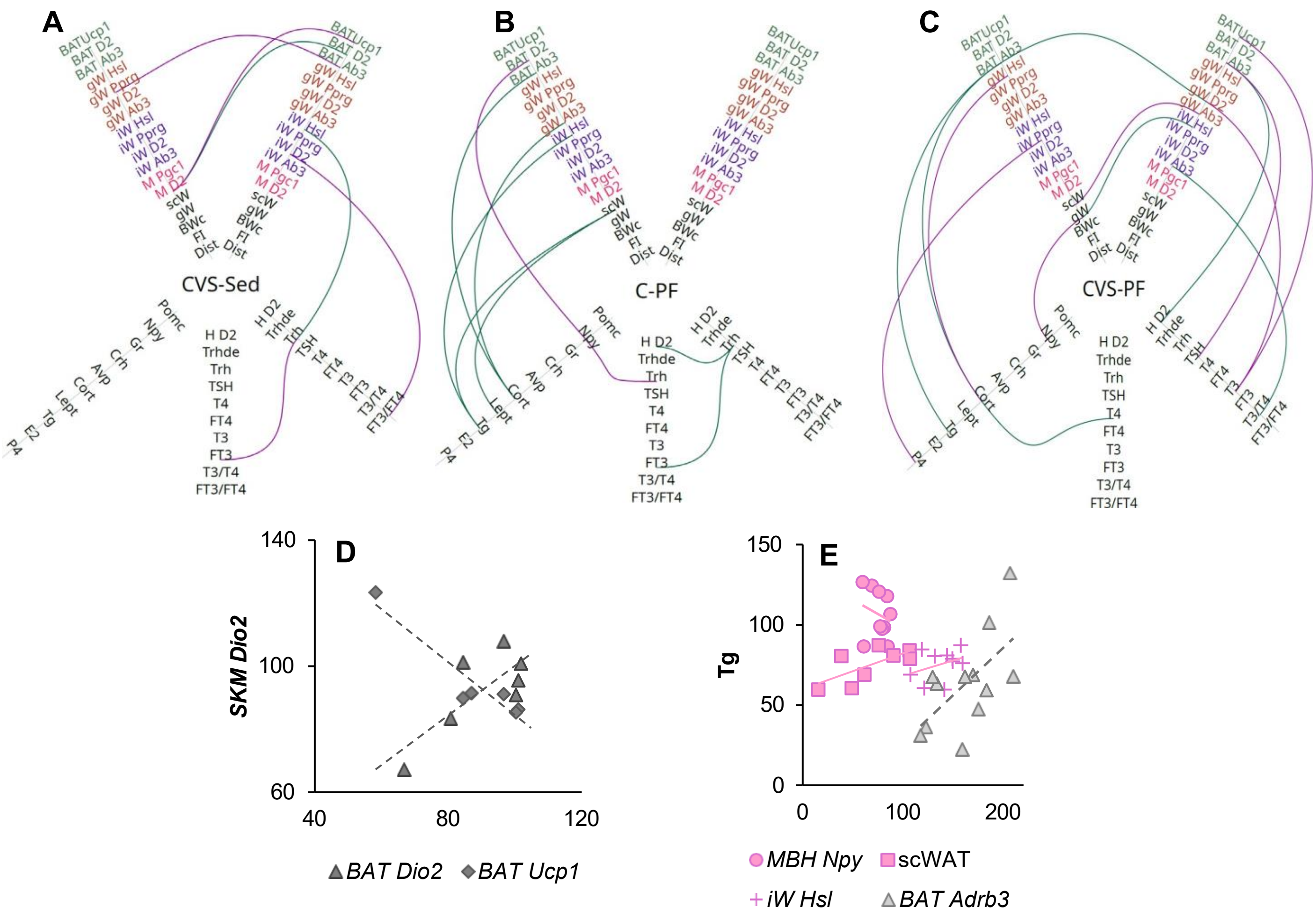

Supplementary Figure S3. Correlations between variables in female rats. (A-C) Hive plot diagrams of correlations among values of gene expressions in hypothalamus and peripheral tissues, and of serum hormone concentrations from pair-fed (PF) control (C) rats or, of previously submitted to chronic variable stress (CVS) female rats. (D) Correlation between skeletal muscle (SKM) Dio2 expression levels and Dio2 and Ucp1 expression in brown adipose tissue (BAT) in CVS-Sed rats. (E) Correlation between serum Tg levels and expression levels of MBH Npy, inguinal WAT (iW) Hsl (Lipe) and interscapular subcutaneous WAT (scWAT) weights in C-PF rats and, BAT Adrb3 expression in CVS-PF female rats.

Males

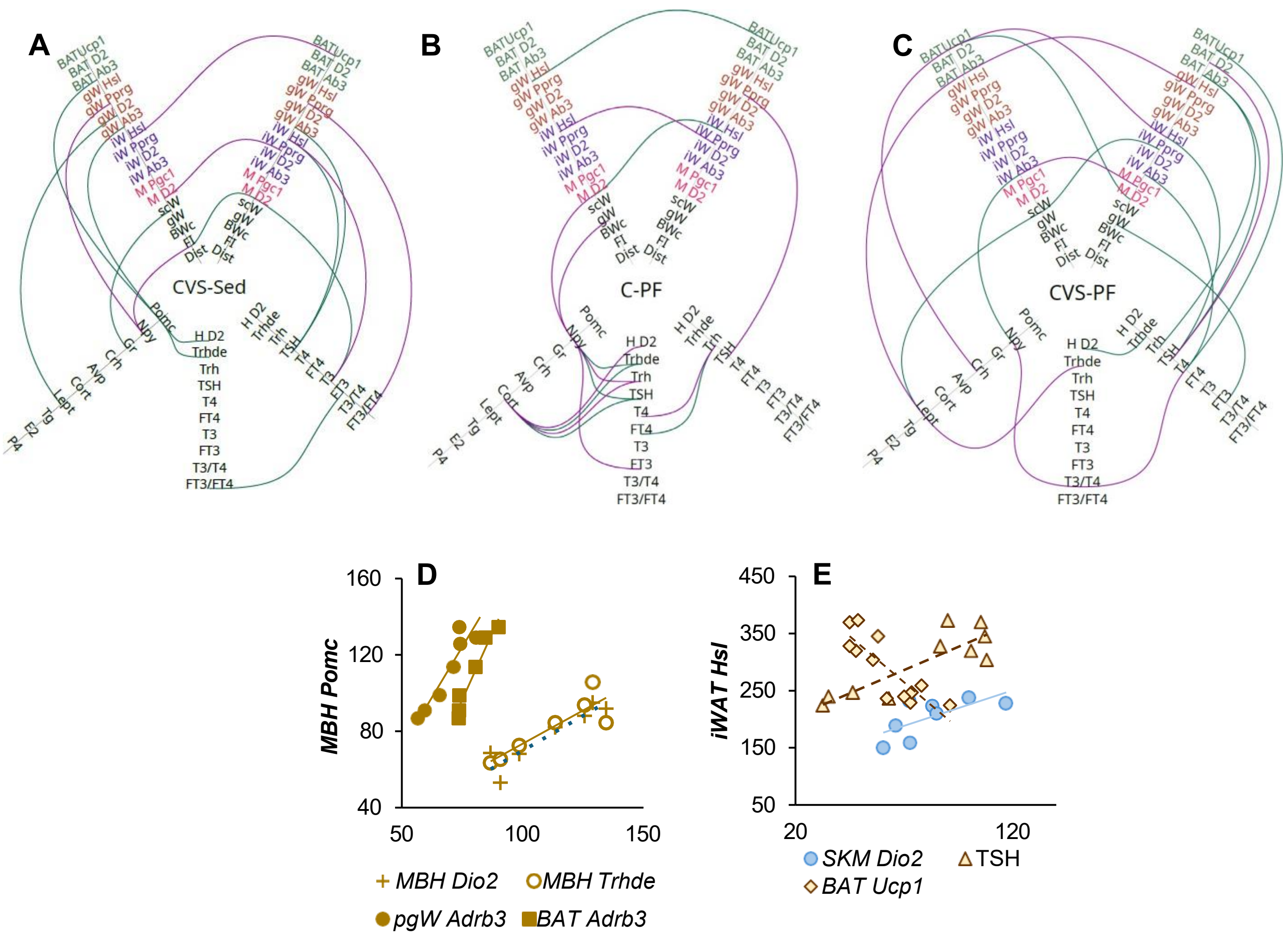

Supplementary Figure S4. Correlations between variables in male rats. (A-C) Hive plot diagrams of correlations among values of gene expressions in hypothalamus and peripheral tissues, and of serum hormone concentrations from pair-fed (PF) control (C) rats or, of previously submitted to chronic variable stress (CVS) male rats. (D) Correlation between the expression levels of MBH Pomc and MBH Dio2 and Trhde, Adrb3 in pgWAT and BAT of CVS-Sed male rats. (E) Correlation between the expression levels of iWAT Hsl (Lipe) and SKM Dio2 in C-PF male and, BAT Ucp1 and serum TSH concentration of CVS-PF male rats.

	PND 31	PND 32	PND 33	PND 34	PND 35	PND 36	PND 37
Morning	TILT ♀-♂<br>**3 h**	Strobe light ♂<br>**3 h**	White noise ♂<br>**3 h**	White noise ♀<br>**3 h**	Elevated platform ♀-♂<br>**15 min**	Fasting ♂<br>**4 h**	Behavioural test (PAS) ♀-♂
Night		Social Isolation ♀<br>**12 h**	Lights on ♀<br>**24 h**	Social isolation ♂<br>**12 h**		Social Isolation ♀<br>**12 h**	
	PND 38	PND 39	PND 40	PND 41	PND 42	PND 43	PND 44
Morning	Cold exposure ♀<br>**(4°C) 1 h**	Cold exposure ♂<br>**(4°C) 1 h**	TILT ♂<br>**3 h**<br>Restraint ♀<br>**1 h**	Strobe light ♀-♂<br>**3h**		White noise ♂<br>**3 h**<br>Fasting ♀<br>**4 h**	Behavioural test (OFT) ♀-♂
Night	Lights on ♂<br>**24 h**	Lights on ♀<br>**24h**			Social isolation ♀-♂<br>**12 h**		
	PND 45	PND 46	PND 47	PND 48	PND 49	PND 50	PND 51
Morning	Fasting ♂<br>**4 h**	White noise ♂<br>**3 h**	Elevated platform ♀-♂<br>**15 min**	Strobe light ♀<br>**3 h**	Restraint ♀-♂<br>**1 h**	TILT ♀-♂<br>**3 h**	Behavioural test (EPM) ♀-♂
Night	Lights on ♀<br>**24 h**	Fasting ♀<br>**12 h**		Lights on ♂<br>**24 h**			
	PND 52	PND 53	PND 54	PND 55	PND 56	PND 57	PND 58
Morning	Cold exposure ♂<br>**(4°C) 1 h**<br>White noise ♀<br>**3 h**	Strobe light ♂<br>**3 h**	Elevated platform ♂<br>**15 min**	TILT ♀<br>**3 h**	Cold exposure ♀<br>**(4°C) 1 h**	Restraint ♂<br>**1 h**<br>Elevated platform ♀<br>**15 min**	Behavioural test (OFT) ♀-♂
Night		Fasting ♀<br>**12 h**	Lights on ♀<br>**24 h**	Fasting ♂<br>**12 h**	Social isolation ♂<br>**12 h**		
	PND 59	PND 60	PND 61	PND 62	PND 63	PND 64	PND 65
Morning		White noise ♂<br>**3 h**<br>**End of stress period** ♀		Elevated platform ♂<br>**15 min**		TILT ♂<br>**3 h**	Behavioural test (EPM) ♂
Night	Lights on ♂<br>**24 h**<br>Social isolation ♀<br>**12 h**		Social isolation ♂<br>**12 h**		Fasting ♂<br>**12 h**		
	PND 66	PND 67	PND 68	PND 69	PND 70		
Morning	Strobe light ♂<br>**3 h**	Cold exposure ♂<br>**(4°C) 1 h**	Restraint ♂<br>**1 h**		**End of stress period** ♂		
Night				Social isolation ♂<br>**12 h**			

Supplementary Table S1. Chronic variable stress (CVS) protocol applied during the adolescent period of male and female rats. Behavioral tests were conducted throughout the CVS exposure period to evaluate stress-induced alterations during adolescence: 1) Spontaneous locomotor activity (SPA) at PND 37 with a photobeam activity system (PAS-Home Cage; San Diego Instruments Co.) [135]; 2) Open field test (OFT) [129], at PND 44 and 58; and 3) Elevated Plus Maze (EPM) [129] at PND 51 in males and females, and PND 65 in males only.

Real-time PCR primers			
**Gene**	**ID**	**Forward**	**Reverse**
Avp	NM_016992.2	TGC CTG CTA CTT CCA GAA CTG C	AGG GGA GAC ACT GTC TCA GCT C
Crh	NM_031019.2	TCT GAG GGA AGT CTT GGA AAT G	GGT ATA TAG GCT CTC TCC CTG T
Ppia	NM_017101.1	GCT GGA CCA AAC ACA AAT GG	CTC CTG AGC TAC AGA AGG AAT G
Nr3c1 (Gr)	NM_012576.2	CCT CAG CGC TCT TGG AAA TTA	CCA CCC TTC TGT CCT GTT TAT G
Pomc	NM_139326.3	TTC ATG ACC TCC GAG AAG AGC	TGT GCG CGT TCT TGA TGA TG
Trh	NM_013046.3	AGA GGG AGA GGG TGT CTT AAT	GCT AGT GAA GGG AAC AGG ATA G
**End-point PCR primers**			
**Gene**	**ID**	**Forward**	**Reverse**
Adrb3	NM_013108.2	TCTGTGCTGGCTGCCCTTCTT	CTTCTCCTCCCCCAACCCTCAA
Dio2	NM_031720.3	GATGCTCCCAATTCCAGTGT	AGGCTGGCAGTTGCCTAGTA
Hprt	NM_012583.2	CCTCAGTCCCAGCGTCGTGA	TGGGGCTGTACTGCTTGACCA
Npy	NM_012614.2	TATCCCTGCTCGTGTGTTTG	GTTCTGGGGGCATTTTCTG
Ppargamma (Pparg)	NM_013124	CCCTTTACCACGGTTGATTTCTC	GCAGGCTCTACTTTGATCGCACT
Ppia	NM_017101.1	CGAGCTGTTTGCAGACAAAGTTCC	GATGGGGTGGGGGTGCTCTC
Ppargc1a (Pgc1a)	NM_031347.1	ATGGAGTGACATAGAGTGTGC	GTGCTAAGACCGCTGCATTC
Pomc	NM_139326.3	GAGATTCTGCTACAGTCGCTC	TTGATGATGGCGTTCTTGAA
Trhde	NM_001108991.1	CTGGATCGCATACAAAAA	GGACAGCCAAATAATTGCT
Ucp1	NM_012682.2	GGATCAAACCCCGCTACACTG	CAGGATCCGAGTCGCAGAAAA
Hsl (Lipe)	NM_012859.1	TCACGCTACATAAAGGCTGCT	CCACCCGTAAAGAGGGAACT

Supplementary Table S2. Primer's GenBank accession number and sequences used for PCR.

	C	CVS
**Males**		
FI (g/day)	23 ± 0.3	23 ± 0.3
BWg (g)	231 ± 3.2	210 ± 3.8*
RFI (g/day/g BW)	67 ± 1.0	70 ± 1.0*
FE (g BWg /100 g food)	29 ± 0.5	27 ± 0.6*
**Females**		
FI (g/day)	17 ± 0.3$^{\sigma}$	18 ± 0.3*$^{\sigma}$
BWg (g)	116 ± 4.5$^{\sigma}$	113 ± 5.2$^{\sigma}$
RFI (g/day/g BW)	79 ± 1.3$^{\sigma}$	85 ± 1.6*$^{\sigma}$
FE (g BWg /100 g food)	25 ± 1.3	23 ± 1.3

Supplementary Table S3. Food intake (FI), body weight gain (BWg), relative food intake (RFI), and food efficiency (FE) during adolescence in control (C) and stressed (CVS) rats. The results are expressed as mean ± S.E.M. and analyzed by two-way ANOVA followed by Tukey's post-hoc test. * P<0.05 vs. C group; σ P<0.05 vs. males of the same group.

	Activity		Stress		Sex		Activity*Stress		Activity*Sex		Stress*Sex		Activity*Stress*Sex	
	F	***P***	***F***	***P***	***F***	***P***	***F***	***P***	***F***	***P***	***F***	***P***	***F***	***P***
**FI**	F(2,129) 108.0	**<0.0001**	F(1,129) 0.5723	0.4507	F(1,129) 3.975	**0.0483**	F(2,129) 1.035	0.3580	F(2,129) 0.8080	0.4480	F(1,129) 6.757	**0.0104**	F(2,129) 1.213	0.3006
**BW**	F(2,129) 67.72	**<0.0001**	F(1,129) 1.361	0.2456	F(1,129) 19.82	**<0.0001**	F(2,129) 0.6152	0.5421	F(2,129) 0.2480	0.7807	F(1,129) 14.19	**0.0003**	F(2,129) 6.112	**0.0029**
**pgWAT**	F(2,118) 21.58	**<0.00001**	F(1,118) 0.93	0.33	F(1,118) 0.55	0.94	F(2,118) 0.051	0.95	F(2,118) 3.33	0.03	F(1,118) 0.9	0.34	F(2,118) 1.66	0.19
**rWAT**	F(2,128) 15.86	**<0.0001**	F(1,128) 0.003	0.95	F(1,128) 1.62	0.2	F(2,128) 1.44	0.23	F(2,128) 1.17	0.31	F(1,128) 0.76	0.38	F(2,128) 3.14	0.04
**iscWAT**	F(2,114) 10.52	**<0.0001**	F(1,114) 0.017	0.89	F(1,114) 3.37	0.06	F(2,114) 0.16	0.84	F(2,114) 0.06	0.93	F(1,114) 0.3	0.58	F(2,114) 1.13	0.32
**Leptin**	F(2,110) 65.82	**<0.0001**	F(1,110) 1.72	0.19	F(1,110) 2.2	0.11	F(2,110) 1.03	0.35	F(2,110) 2.22	0.11	F(1,110) 3.22	0.0.07	F(2,110) 0.71	0.49
**Tg**	F(2,113) 25.59	**<0.0001**	F(1,113) 0.04	0.82	F(1,113) 10.74	**0.001**	F(2,113) 2.05	0.13	F(2,113) 21.04	**<0.0001**	F(1,113) 2.22	0.13	F(2,113) 1.37	0.25
**PVN *Crh***	F(2,104) 5.59	**0.004**	F(1,104) 1.9	0.17	F(1,104) 0.005	0.93	F(2,104) 1.74	0.17	F(2,104) 0.43	0.64	F(1,104) 7.26	**0.008**	F(2,104) 3.5	**0.03**
**PVN *Avp***	F(2,52) 17.92	**<0.0001**	F(1,52) 46.88	**<0.0001**	F(1,52) 1.63	0.2	F(2,52) 4.69	0.013	F(2,52) 1.75	0.18	F(1,52) 0.03	0.85	F(2,52) 11.07	**<0.0001**
**PVN *Gr***	F(2,96) 3.835	**0.0250**	F(1,96) 14.95	**0.0002**	F(1,96) 23.27	**<0.0001**	F(2,96) 1.802	0.1704	F(2,96) 0.6403	0.5294	F(1,96) 8.802	**0.0038**	F(2,96) 1.329	0.2696
**Cort**	F(2,104) 109.8	**<0.0001**	F(1,104) 82.14	**<0.0001**	F(1,104) 59.17	**<0.0001**	F(2,104) 59.19	**<0.0001**	F(2,104) 11.11	**<0.0001**	F(1,104) 0.83	0.36	F(2,104) 9.8	**0.0001**
**MBH *Pomc***	F(2,98) 189.8	**<0.0001**	F(1,98) 144.2	**<0.0001**	F(1,98) 2.16	0.144	F(2,98) 120.7	**<0.0001**	F(2,98) 3.36	**0.038**	F(1,98) 0.4624	0.49	F(2,98) 2.14	0.122
**MBH *Npy***	F(2,104) 18.82	**<0.0001**	F(1,104) 45.88	**<0.0001**	F(1,104) 66.87	**<0.0001**	F(2,104) 2.28	0.106	F(2,104) 0.617	0.541	F(1,104) 61.74	**<0.0001**	F(2,104) 3.237	**0.043**
**MBH *Dio2***	F(2,105) 18.85	**<0.0001**	F(1,105) 120.5	**<0.0001**	F(1,105) 1.09	0.29	F(2,105) 21.45	**<0.0001**	F(2,105) 2.01	0.12	F(1,105) 0.0017	0.96	F(2,105) 0.86	0.42
**MBH *Trhde***	F(2,109) 1.21	0.3	F(1,109) 0.11	0.73	F(1,109) 10.56	**0.0011**	F(2,109) 3.72	**0.027**	F(2,109) 1.36	0.25	F(1,109) 3.59	0.06	F(2,109) 11.45	**<0.0001**
**PVN *Trh***	F(2,104) 22.5	**<0.0001**	F(1,104) 39.13	**<0.0001**	F(1,104) 10.46	**0.001**	F(2,104) 6.77	**0.001**	F(2,104) 7.79	**0.0007**	F(1,104) 18.84	**<0.0001**	F(2,104) 8.65	**0.0003**
**TSH**	F(2,115) 12.03	**<0.0001**	F(1,115) 0.09	0.76	F(1,15) 0.44	0.5	F(2,115) 1.36	0.25	F(2,115) 0.55	0.57	F(1,115) 0.26	0.61	F(2,115) 0.7	0.49
**T4**	F(2,113) 3.4	**0.03**	F(1,113) 4.72	**0.03**	F(1,113) 1.14	0.28	F(2,113) 0.6	0.54	F(2,113) 0.03	0.96	F(1,113) 2.86	0.09	F(2,113) 2.1	0.12
**T3**	F(2,117) 2.01	0.13	F(1,117) 12.56	**0.0006**	F(1,117) 49.23	**<0.0001**	F(2,117) 0.2	0.81	F(2,117) 2.37	0.09	F(1,117) 10.69	**0.001**	F(2,117) 1.58	0.2
**fT4**	F(2,58) 0.2	0.81	F(1,58) 0.74	0.39	F(1,58) 11.36	**0.001**	F(2,58) 0.58	0.56	F(2,58) 0.13	0.87	F(1,58) 2.85	0.09	F(2,58) 0.7	0.49
**fT3**	F(2,54) 2.58	0.08	F(1,54) 19.25	**<0.0001**	F(1,54) 102.9	**<0.0001**	F(2,54) 0.67	0.51	F(2,54) 4.77	**0.012**	F(1,54) 40.31	**<0.0001**	F(2,54) 0.7	0.49

Supplementary Table S4A. Three-way ANOVA of the effects of CVS during adolescence, voluntary exercise, and sex.

	Activity		Stress		Sex		Activity*Stress		Activity*Sex		Stress*Sex		Activity*Stress*Sex	
	F	***P***	***F***	***P***	***F***	***P***	***F***	***P***	***F***	***P***	***F***	***P***	***F***	***P***
**fT3/fT4**	F(2,53) 1.49	0.23	F(1,53) 10.29	**0.002**	F(1,53) 15.01	**0.0003**	F(2,53) 0.75	0.47	F(2,53) 2.56	0.08	F(1,53) 9.01	**0.004**	F(2,53) 1.5	0.23
**T3/T4**	F(2,104) 0.71	0.49	F(1,104) 0.23	0.63	F(1,104) 31.37	**<0.0001**	F(2,104) 1.87	0.15	F(2,104) 0.6	0.54	F(1,104) 0.81	0.36	F(2,104) 2.89	0.06
**SM *Dio2***	F(2,98) 62.51	**<0.0001**	F(1,98) 86.62	**<0.0001**	F(1,98) 14.02	**0.0003**	F(2,98) 17.81	**<0.0001**	F(2,98) 0.52	0.59	F(1,98) 0.205	0.65	F(2,98) 6.24	**0.002**
**SM *Pgc1a***	F(2,101) 59.75	**<0.0001**	F(1,101) 38.53	**<0.0001**	F(1,101) 0.86	0.35	F(2,101) 39.47	**<0.0001**	F(2,101) 0.27	0.76	F(1,101) 7.09	**0.0090**	F(2,101) 5.01	**0.008**
**pgW *Adrb3***	F(2,118) 253.8	**<0.0001**	F(1,118) 136.8	**<0.0001**	F(1,118) 7.849	**0.005**	F(2,118) 11.88	**<0.0001**	F(2,118) 0.16	0.84	F(1,118) 3.27	0.073	F(2,118) 1.23	0.29
**pgW *Dio2***	F(2,119) 427.8	**<0.0001**	F(1,119) 145.2	**<0.0001**	F(1,119) 16.14	**0.0001**	F(2,119) 29.66	**<0.0001**	F(2,119) 8.66	**0.0003**	F(1,119) 0.18	0.67	F(2,119) 0.036	0.96
**pgW *Pparg***	F(2,118) 199.0	**<0.0001**	F(1,118) 98.97	**<0.0001**	F(1,118) 3.008	0.0855	F(2,118) 85.97	**<0.0001**	F(2,118) 8.43	**0.0004**	F(1,118) 4.19	**0.04**	F(2,118) 6.47	**0.002**
**pgW *Hsl (Lipe)***	F(2,115) 164.3	**<0.0001**	F(1,115) 1658	**<0.0001**	F(1,115) 183.6	**<0.0001**	F(2,115) 91.38	**<0.0001**	F(2,115) 17.55	**<0.001**	F(1,115) 1.28	0.26	F(2,115)14.1	**<0.001**
**iW *Adrb3***	F(2,60) 104.7	**<0.0001**	F(1,60) 357.0	**<0.0001**	F(1,60) 4.814	**0.032**	F(2,60) 52.29	**<0.0001**	F(2,60) 4.652	**0.013**	F(1,60) 15.59	**0.0002**	F(2,60) 8.2	**0.0007**
**iW *Dio2***	F(2,60) 473.9	**<0.0001**	F(1,60) 159.5	**<0.0001**	F(1,60) 43.38	**<0.0001**	F(2,60) 49.88	**<0.0001**	F(2,60) 84.68	**<0.0001**	F(1,60) 18.59	**<0.0001**	F(2,60) 7.25	**0.0015**
**iW *Pparg***	F(2,113) 47.88	**<0.0001**	F(1,113) 33.54	**<0.0001**	F(1,113) 10.74	**0.001**	F(2,113) 36.24	**<0.0001**	F(2,113) 3.45	0.22	F(1,113) 1.46	0.22	F(2,113) 2.11	0.12
**iW *Hsl (Lipe)***	F(2,116) 122.7	**<0.0001**	F(1,116) 94.87	**<0.001**	F(1,116) 126.7	**<0.001**	F(2,116) 8.67	**<0.0001**	F(2,116) 18.68	**<0.0001**	F(1,116) 8.2	**0.005**	F(2,116) 0.44	0.63
**BAT *Adrb3***	F(2,102) 10.99	**<0.0001**	F(1,102) 3.411	0.067	F(1,102) 36.51	**<0.0001**	F(2,102) 11.32	**<0.0001**	F(2,102) 6.05	**<0.0001**	F(1,102) 0.901	0.34	F(2,102) 6.26	**0.0027**
**BAT *Dio2***	F(2,98) 49.8	**<0.0001**	F(1,98) 0.33	0.56	F(1,98) 2.45	0.12	F(2,98) 2.01	0.13	F(2,98) 10.39	**<0.0001**	F(1,98) 1.02	0.31	F(2,98) 7.89	**0.0010**
**BAT *Ucp1***	F(2,96) 34.12	**<0.0001**	F(1,96) 109.2	**<0.0001**	F(1,96) 67.01	**<0.0001**	F(2,96) 44.53	**<0.0001**	F(2,96) 7.470	**0.0010**	F(1,96) 2.329	0.1303	F(2,96) 4.702	**0.0007**

Supplementary Table S4B. Three-way ANOVA of the effects of CVS during adolescence, voluntary exercise, and sex (continuation).

	Activity		Stress		Activity*Stress	
	F	***P***	***F***	***P***	***F***	***P***
**E2**	F(2,62) 0.51	0.6	F(1,62) 0.58	0.56	F(2,62) 0.06	0.79
**P4**	F(2,65) 0.16	0.84	F(1,65) 7.6	**0.001**	F(2,65) 0.81	0.36
**PRL**	F(2,123) 1.023	0.36	F(1,123) 0.47	0.49	F(2,123) 0.95	0.38

Supplementary Table S4C. Two-way ANOVA of the effect of CVS during adolescence and voluntary exercise on estradiol and progesterone levels.
